# Pathophysiological Consequences of Neuronal α-Synuclein Overexpression: Impacts on Ion Homeostasis, Stress Signaling, Mitochondrial Integrity, and Electrical Activity

**DOI:** 10.3389/fnmol.2018.00049

**Published:** 2018-03-07

**Authors:** Johan Tolö, Grit Taschenberger, Kristian Leite, Markus A. Stahlberg, Gesche Spehlbrink, Janina Kues, Francesca Munari, Stefano Capaldi, Stefan Becker, Markus Zweckstetter, Camin Dean, Mathias Bähr, Sebastian Kügler

**Affiliations:** ^1^Department of Physiology, The Sahlgrenska Academy at Gothenburg University, Gothenburg, Sweden; ^2^Department of Neurology, University Medical Center Goettingen, Göttingen, Germany; ^3^Center Nanoscale Microscopy and Physiology of the Brain, Göttingen, Germany; ^4^European Neuroscience Institute, Department of Transsynaptic Signaling, Göttingen, Germany; ^5^German Center for Neurodegenerative Diseases, Göttingen, Germany; ^6^Department for NMR-based Structural Biology, Max Planck Institute for Biophysical Chemistry, Göttingen, Germany; ^7^Biocrystallography Laboratory, Department of Biotechnology, University of Verona, Verona, Italy

**Keywords:** synuclein, ATP, calcium, reactive oxygen species, thiol oxidation, apoptosis, Bcl-Xl, synchronized network activity

## Abstract

α-Synuclein (α-Syn) is intimately linked to the etiology of Parkinson's Disease, as mutations and even subtle increases in gene dosage result in early onset of the disease. However, how this protein causes neuronal dysfunction and neurodegeneration is incompletely understood. We thus examined a comprehensive range of physiological parameters in cultured rat primary neurons overexpressing α-Syn at levels causing a slowly progressive neurodegeneration. In contradiction to earlier reports from non-neuronal assay systems we demonstrate that α-Syn does not interfere with essential ion handling capacities, mitochondrial capability of ATP production or basic electro-physiological properties like resting membrane potential or the general ability to generate action potentials. α-Syn also does not activate canonical stress kinase Signaling converging on SAPK/Jun, p38 MAPK or Erk kinases. Causative for α-Syn-induced neurodegeneration are mitochondrial thiol oxidation and activation of caspases downstream of mitochondrial outer membrane permeabilization, leading to apoptosis-like cell death execution with some unusual aspects. We also aimed to elucidate neuroprotective strategies counteracting the pathophysiological processes caused by α-Syn. Neurotrophic factors, calpain inhibition and increased lysosomal protease capacity showed no protective effects against α-Syn overexpression. In contrast, the major watchdog of outer mitochondrial membrane integrity, Bcl-Xl, was capable of almost completely preventing neuron death, but did not prevent mitochondrial thiol oxidation. Importantly, independent from the quite mono-causal induction of neurotoxicity, α-Syn causes diminished excitability of neurons by external stimuli and robust impairments in endogenous neuronal network activity by decreasing the frequency of action potentials generated without external stimulation. This latter finding suggests that α-Syn can induce neuronal dysfunction independent from its induction of neurotoxicity and might serve as an explanation for functional deficits that precede neuronal cell loss in synucleopathies like Parkinson's disease or dementia with Lewy bodies.

## Introduction

The small presynaptic protein α-Synuclein (α-Syn) is highly abundant in human brain but its exact function remains enigmatic. α-Syn is linked to the etiology of Parkinson's disease, a common and mostly idiopathic neurodegenerative disorder of the elderly. In rare genetic cases multiplications of the *SNCA* gene or increased promoter activities cause early onset PD. This suggested that increased α-Syn levels might be directly causative for PD. α-Syn is a core component of aggregated protein species found in brains of almost all patients suffering from PD, but also in brains of patients with Alzheimer's disease (AD), Dementia with Lewy Bodies (DLB) and Multiple Systems Atrophy (MSA). These aggregates termed Lewy bodies/Lewy neurites are found all over the patients' brains, not only in those brain nuclei responsible for the motor malfunctions of PD. However, whether α-Syn aggregation is cause, consequence or coincidence of neuronal malfunctions in PD and related disorders is still unclear, as are the mechanisms by which α-Syn contributes to this neurodegeneration (Del Tredici and Braak, 2016; Dettmer et al., 2016).

During the past years a wealth of studies have addressed potential α-Syn-related disease mechanisms, implicating mitochondrial/respiratory chain dysfunctions, impaired protein degradation, impaired ion handling capabilities, reactive oxygen species (ROS), among many others, as causative for onset and/or progression of PD. The vast majority of these studies have been performed in experimental systems that are easy to manipulate genetically, in order to achieve the levels of α-Syn overexpression necessary to induce phenotypes. Although much could be learned from Drosophila-, C-elegans-, and yeast-based research, it should be noted that these systems do not have endogenous α-Syn orthologs, meaning that for them α-Syn is an unknown protein. Overexpressing α-Syn in cell lines or in cells derived from brain cells which do not recapitulate features of excitable neurons might also generate false-positive or false-negative results, due to divergent physiological functions present in neurons vs. other cell types, such as terminal differentiation and inability to divide, capabilities to handle ion fluxes, relative distribution of cytoplasmic volume between peri-nuclear and neuritic spaces, and energy demands at pre- and postsynaptic sites far remote from the cell body (Buchman and Ninkina, 2008; Gubellini and Kachidian, 2015).

Given that synucleinopathies like PD or DLB mostly affect neurons, it is justified to elucidate pathological mechanisms caused by α-Syn in mammalian neurons. Furthermore, it is important to address interactions and succession of α-Syn-caused dysfunctions in order to be able to identify potential therapeutic targets. The experimental system used for this study consists of a neuron-glia co-culture derived from E18 rat brains (Kügler et al., 2001; Taschenberger et al., 2013). Without need for cell culture medium replacements these cultures survive for >4 weeks, due to astroglial support of neuronal maintenance. Neurons grow dense neuritic networks, fasciculate axons, and show endogenous, non-stimulated electrical activity (Murphy et al., 1992). Synucleins overexpressed specifically in neurons by means of AAV viral vectors under control of the neuron-specific synapsin 1 gene promoter (Kügler et al., 2003) are partially secreted into cell culture supernatant, form intracellular proteinase K resistant aggregates, substantially impact on mitochondrial morphology, and, depending on expression level, start to show signs of synuclein-dependent degeneration after about 6–8 days of overexpression (Karpinar et al., 2009; Taschenberger et al., 2012, 2013). Similar to results obtained in the substantia nigra of rodents *in vivo*, β-Syn is somewhat less neurotoxic than α-Syn in this model, while γ-Syn does not cause any neuron loss over time and thus can serve as a valid control (Taschenberger et al., 2013).

In several experiments of this study, α-Syn was expressed together with genetically encoded fluorescent sensor proteins, thereby allowing to record physiological signals from living cells in real time. Our results demonstrate that α-Syn levels sufficient to induce a slowly progressive neuronal death do not impair important physiological functions, such as Ca^2+^ handling, ATP production, mitochondrial or cell membrane potential, generation of cytoplasmic ROS or activation of canonical stress kinase pathways. However, α-Syn caused a pathological sequence of mitochondrial deformation, increased mitochondrial thiol oxidation, mitochondrial outer membrane permeabilization (MOMP), and caspase activation to execute an apoptosis-like cell death. Blocking this cell death allowed to record the substantial inhibition of endogenous neuronal network activity caused by α-Syn in aged neurons, that might serve as an explanation for neuronal dysfunctions in synucleinopathies even in absence of evident neuron loss.

## Materials and methods

### Neuronal cell culture

Primary cortical neurons were prepared from E18 rat pups as described (Kügler et al., 2001). Neurons were seeded in 24-well plates at a density of 250,000 cells per well. Cell culture media were not replaced or exchanged during the course of the study up to the time point when cells were imaged in artificial cerebrospinal fluid (aCSF; 128 mM NaCl, 3 mM KCl, 1 mM MgSO_4_, 1 mM NaH_2_PO_4_, 10 mM glucose, 30 mM HEPES). For high-K^+^ buffer, NaCl was reduced to 68 mM and KCl increased to 63 mM. All experimental animal procedures were conducted according to approved experimental animal licenses (33.9-42502-04-11/0408) issued by the responsible animal welfare authority (Niedersächsisches Landesamt für Verbraucherschutz und Lebensmittelsicherheit) and controlled by the local animal welfare committee of the University Medical Center Göttingen. This neuronal cell culture system was chosen due to its relative ease of preparation and robust long-term maintenance capabilities. It has to be noted that these cells do not reflect the dopaminergic neurotransmitter phenotype of neurons of the substantia nigra pars compacta, which are especially prone to neurodegeneration in PD. However, synucleinopathies like PD and DLB affect a wide variety of neuronal subtypes during disease progression, and thus primary neurons from rat cortex can be considered as a valid neuronal model system. In addition, these neurons possess electrical pacemaker activities closely resembling that of nigral dopaminergic neurons.

### AAV vectors

Recombinant adeno-associated viral vectors of serotype 6 (AAV-6) were prepared by transient transfection of vector genome plasmids (for details see Supplemental Figure 1) with the DP6 helper plasmid in HEK293 cells, viral particles were purified from cell lysates by iodixanol gradient centrifugation and heparin affinity chromatography. After extensive dialysis against PBS particles were frozen in single use aliquots at −80°C. Genome titres were determined by qPCR and >98% purity was confirmed by SDS-PAGE. Vector genome (vg) titre vs. transducing units (tu) titre was determined with EGFP expressing vectors in primary neurons and was estimated to be 1:30 (tu:vg). All constructs expressed transgenes from a neuron-specific synapsin1 gene promoter.

### Synuclein expression levels

All experiments of this study were conducted by expression of human wild-type α-, β- or γ-synucleins (α-, β- or γ-Syn). Although mutations in synucleins might differentially impact on the investigated parameters, they represent extremely rare familial cases and were thus not considered here. All experiments were conducted by synuclein gene transfer with 1 × 10e8 tu of the respective AAV-6 vector per 250,000 neurons if not otherwise stated. The level of overexpression achieved by this mode was determined by expressing rat α-Syn by different titres of AAV and detecting the target protein with antibodies specific for rat α-Syn. This procedure is necessary as for no human α-Syn-specific antibody it is proven that it detects the rat α-Syn, which is endogenously present in the rat neurons, with the same sensitivity as the human α-Syn, which is overexpressed in the neurons. Using this approach we found that 1 × 10e7 and 3 × 10e7 tu of AAV-α-Syn(rat) resulted in about 10–13-fold higher α-Syn levels as compared to endogenous levels, while titres of 1 × 10e8 and 3 × 10e8 resulted in 17–20-fold higher α-Syn levels as compared to endogenous levels. Using the same range of AAV titres with human α-Syn and respective antibodies, the increase of protein expression level with 3-fold increments in titre was 2-fold each, but could not be related to the levels of endogenous rat α-Syn. Importantly, in both cases, the increase of protein expression from 3 × 10e7 tu to 1 × 10e8 tu was roughly 2-fold. Notably, α-Syn expressed from AAV vectors at 3 × 10e7 tu did not cause significant cell death during the lifetime of the neuronal cultures (3–4 weeks), while α-Syn expressed from AAV vectors at 1 × 10e8 tu caused significant degeneration after about 2 weeks in culture.

### Imaging setup

All recording of fluorescent signals from genetically encoded sensors (GES) were performed on a Zeiss Examiner upright microscope stand (Zeiss GmbH) equipped with RC-26GLP imaging chamber, PH-1 heating platform, TC-344C heating controller, SHM-8 in-line heater (Warner Instruments), W Plan-Apochromat 20x/1.0 objective, W Plan-Apochromat 63x/1.0 objective, Colibri LED excitation light source and two Zeiss MRm cameras (Zeiss GmbH) set up for dual wavelength imaging. Cell cultures were continually superfused during imaging with solutions warmed to 34°C. A VC-8T valve control system (Warner Instruments) together with a custom built computer interface was used to switch between up to eight different solutions.

### Field stimulation

Field stimulation was performed using a SIU-102 stimulus isolation unit (Warner Instruments) together with a custom built function generator based on an Arduino microcontroller board (Arduino). Electrodes were submerged in imaging medium and separated by 15 mm during imaging. The SIU-102 was set to constant current (100 mA) and bi-polar pulse mode (500 μs pulse width).

### Imaging of free-Ca^2+^

Ca^2+^ imaging was performed essentially as described (Mironov et al., 2009), using the red fluorescent genetically encoded calcium indicator (GECI) RCaMP1e (Akerboom et al., 2013) as a sensor. RCaMP1e was excited by light from a 590 nm LED (Colibri, Zeiss) and passing through a 60 HE (Colibri, Zeiss) filter cube. Recording of free-Ca^2+^ from the mitochondrial matrix was enabled through fusion of RCaMP1e with the targeting sequence of subunit VIII of cytochrome c oxidase to create mt-RCaMP1e. Throughout the article we report Δ*F*/*F*_0_ values rather than converted to actual calcium concentrations.

RCaMP1e and variants were described recently (Akerboom et al., 2013). There its *K*_d_ was measured to be 1.6 μM *in vitro* with a Hill coefficient of 3.4 and its *pK*_a_ as 5.9. Many sources report resting cytosolic free-Ca^2+^ levels within neurons to be kept at very low concentrations of around 100 nM (Ross, 1989; Marambaud et al., 2009). The resting concentrations of free-Ca^2+^ within the mitochondrial matrix have been more difficult to determine but are reported to be similar to those within the cytosol (Babcock and Hille, 1998). The pH within the mitochondria is generally accepted to be higher than that within the cytosol. However, Akerboom et al. (2013) demonstrates that the difference in response of RCaMP1f with pH between 7-8 is negligible. Since the pKa of RCaMP1e is similar to that of RCaMP1f, it can be assumed that this holds also for this variant of the sensor.

Using the *in vitro* values for *K*_*d*_ and Hill coefficient, the theoretical response curve of RCaMP1e looks like the following: 
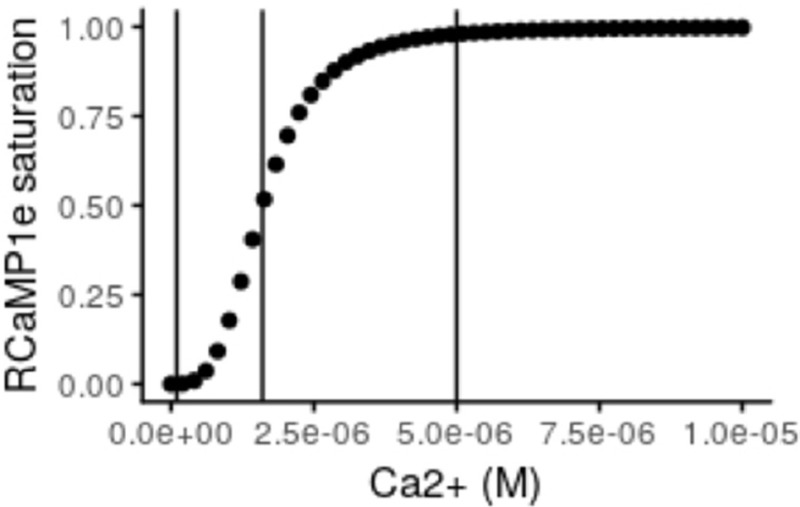
 where the three vertical lines, from left to right, indicate approximate resting free-Ca^2+^, *K*_*d*_ and resting level multiplied with 50 respectively. This shows that when the neuron is at rest, the fraction of sensor molecules that bind calcium is very low and in a region where small fluctuations of free-Ca^2+^ will not produce a detectable response. On the other hand, during activity, the concentration of free-Ca^2+^ has been reported to increase up to 100 fold and even higher within the mitochondria (Babcock and Hille, 1998; Marambaud et al., 2009) which would be levels sufficient to completely saturate RCaMP1e. However, stimulating neurons in culture electrically at 10 Hz did not reach such levels in our experiments. KCl application on the other hand, sometimes caused increases in cytosolic as well as mitochondrial free-Ca^2+^ levels high enough to saturate RCaMP1e.

### Imaging of ATP

Imaging of intracellular ATP concentrations was performed essentially as described (Toloe et al., 2014) using the GES ATeam1.03 (Imamura et al., 2009) with one exception. It was not necessary to calculate the rate of ATP re-generation after depletion using FCCP based on the rate of depletion, instead it was measured directly from the slope of the curve. The CFP/YFP based ratiometric FRET-sensor ATeam1.03 was excited using a 455 nm LED (Colibri, Zeiss) together with the 78 HE (Zeiss) filter set. Dual wavelength detection followed using two spatially aligned MRm cameras.

ATP concentrations were estimated by using the min level FRET ratio after depletion of ATP by decoupling the mitochondria using FCCP as previously described (Toloe et al., 2014). The equation:

$$\begin{array}{l} R=R_{\text{min}}+\frac{\left(R_{\text{max}}-R_{\text{min}}\right){\left[\text{ATP}\right]}^{2}}{K_{\text{d}}^{2}+{\left[\text{ATP}\right]}^{2}} \end{array}$$

was rearranged into:

$$\begin{array}{l} \left[\text{ATP}\right]=K_{\text{d}}\sqrt{\frac{1-r}{R_{\text{max}}/R_{\text{min}}-r}} \end{array}$$

where *K*_d_ is the dissociation constant and *R*_max_/*R*_min_ the dynamic range of the FRET ratio of the sensor. *R*/*R*_min_ has been substituted for *r*, representing the relative FRET ratio (change from min level). For simplicity, this assumes a Hill-coefficient of 2 (2.1 according to Imamura et al Imamura et al., 2009). These authors published *in vitro* values for *K*_d_ and R_max_/*R*_min_ of 3.3 mM and 2.3 respectively which fits well with values determined experimentally in cell by us and reported previously (Toloe et al., 2014).

### Imaging of redox potential

Imaging of redox potential from the cytosol and mitochondrial matrix was performed essentially as described (Hanson et al., 2004; Grosser et al., 2012) using roGFP1 and mt-roGFP1. roGFP1 is a ratiometric sensor and sequential excitation was done using a 365 and 455 nm LED respectively (Colibri, Zeiss) together with a 60 HE (Colibri, Zeiss) filter cube with the excitation filter removed.

The biochemical properties of the redox sensitive genetically encoded sensor roGFP1 have been described (Hanson et al., 2004). All experiments using the roGFP1 sensor done for this publication included a calibration step. After measurements, the cell cultures were left in the imaging chamber and first superfused with 10 mM DTT to completely reduce the sensor. After reaching a stable baseline, the DTT was washed out and replaced with 10 mM H_2_O_2_ to completely oxidize the sensor. This allowed us to calculate the fraction of reduced sensor molecules at any one time according to Equation (5) in Hanson et al. (2004):

$$\begin{array}{l} R=\frac{F-F_{\text{ox}}}{F_{\text{red}}-F_{\text{ox}}} \end{array}$$

where *R* is the fraction, *F* the current fluorescence ratio and *F*_red_ and *F*_ox_ the maximum reduced and oxidized fluorescence ratios respectively. *R* for roGFP1 is related to the oxidation state of DTT through Equation (2) in Hanson et al. (2004):

$$\begin{array}{l} R=\frac{\left[{\text{DTT}}_{\text{red}}\right]/\left[\text{DT}{\text{T}}_{\text{ox}}\right]}{K_{\text{eq}}+\left[\text{DT}{\text{T}}_{\text{red}}\right]/\left[\text{DT}{\text{T}}_{\text{ox}}\right]} \end{array}$$

where *K*_eq_ is the equilibration constant. Hanson et al. (2004) determined *K*_eq_ by plotting *R* against [*DT*T_red_]/[*DT*T_ox_] and fitting the data from a titration curve according to equation 2. They found *K*_eq_ to be 0.070 for roGFP1 *in vitro*. Using the Nernst equation,

$$\begin{array}{l} E_{0\left(roGFP1\right)}^{\prime }=E_{0\left(DTT\right)}^{\prime }-\frac{RT}{\text{nF}}\cdot \text{ln}K_{\text{eq}} \end{array}$$

where *R* is the gas constant (8.315JK^−1^mol^−1^), *T* the absolute temperature (303.15°C), *n* the number of transferred electrons (2) and *F* is the Faraday constant (9.649exp−4Cmol^−1^), Hanson et al. (2004) calculated the equilibrium redox potential for roGFP1 ($E_{0\left(roGFP1\right)}^{\prime }$) at pH 7 and 30°C to be −0.288V. A month later the same group published another study (Dooley et al., 2004) where they calculated the redox potential to be −0.295V and they suggest to use an average value of −0.291V for future work. However, the difference seem to stem from using two different values of $E_{0\left(DTT\right)}^{\prime }$ in the Ernst equation. Hanson et al. (2004) used a value of −0.323V and Dooley et al. (2004) a value of −0.330V. For this work, a value of −0.323V was used.

To calculate the baseline redox potential of the cytosol and mitochondria of neurons using roGFP1, Equation (2) was rearranged into:

$$\begin{array}{l} \frac{K_{\text{eq}}R}{1-R}=\frac{\left[DT{\text{T}}_{\text{red}}\right]}{\left[\text{DT}{\text{T}}_{\text{ox}}\right]} \end{array}$$

and this expression was plugged into the Ernst equation to give:

$$\begin{array}{l} E_{\text{cyt}}^{\prime }=E_{0\left(\text{DTT}\right)}^{\prime }-\frac{RT}{\text{nF}}\cdot \ln\frac{K_{\text{eq}}R}{1-R} \end{array}$$

for calculating the redox potential in the cytosol. For calculating the redox potential within the mitochondrial matrix using mts-roGFP1, the Nernst equation was adapted as in Hanson et al. (2004) to account for the higher pH (7.98) within this compartment according to the following relation:

$$\begin{array}{l} {E^{\prime }}_{0}^{\text{pH}}=E_{0}^{\prime }-60.1\text{mV}\cdot \left(\text{pH}-7\right) \end{array}$$

### Imaging of mitochondrial membrane potential

Relative levels of mitochondrial membrane potential were estimated using the potentiometric fluorescent dye Tetramethylrhodamine-methylesther (TMRM) essentially as described (Ehrenberg et al., 1988; Loew et al., 1993). In short, 6 or 13 DPT with synucleins co-expressed with EGFP, neuronal cultures were labeled with 5 nM TMRM (Life technologies) in aCSF-Hepes for 30 min after which they were transfered to the imaging chamber and continually superfused with aCSF-Hepes containing 2.5 nM TMRM. At first the EGFP signal was recorded using a 455 nM LED. Initial baseline fluorescence intensity from TMRM was then recorded using 590 nm LED illumination for at least 10 frames after which 2 μM FCCP was added to the superfusion media. This resulted in a decline of fluorescence intensity from mitochondria and simultaneous increase in surrounding areas. Both the TMRM and EGFP channels were recorded using a 60 HE (Colibri, Zeiss) filter cube. The data was evaluated by drawing pairs of regions of interest (ROI) around individual mitochondria that co-localized with EGFP within the soma and regions within the cytosol adjacent to these mitochondria. The ratio between the mean fluorescence intensity of the pair of ROI was used as the measure for relative mitochondrial membrane potential.

### Electrophysiology

Whole-cell patch-clamp recordings were performed on 6 or 13 days post transduction (dpt) rat cortical neurons expressing either alpha- or gamma-synuclein, identified by EGFP fluorescence on a Zeiss Examiner.D1 microscope equipped with a 40x W Plan-Apochromat objective and a Colibri LED light-source. Cells on 12.5 mm coverslips were transferred to pre-heated 34°C extracellular solution containing (in mM): 127 NaCl, 3 KCl, 1 MgSO_4_, 1 NaH_2_PO_4_, 30 HEPES, 1.5 CaCl_2_, 10 D(+)-glucose (all from Roth; mOsm = 300, pH = 7.4) including 50 μM APV (Abcam ab12003), 10 μM CNQX (Sigma C239), and 10 μM gabazine (Abcam ab120042). Solution was maintained at 34°C using a TC-20 temperature controller (npi electronic) connected to a heat resistor in the imaging chamber and a heated HPT-2 perfusion tube (npi electronic). Patch pipettes with a resistance of 3-5 MΩ were made from fire polished borosilicate capillaries (Harvard Apparatus, Holliston, U.S.A.; cat. no. 300060, OD 1.5 × ID 0.86 mm) using a horizontal P-97 Micropipette Puller equipped with a 3 × 3 mm box filament (Sutter Instruments Co.; cat. no. P-97 & FB330B). Silver wire electrodes were chlorinated using an ACL-01 apparatus (npi electronic) and a 2 M KCl solution. Patch pipettes were back-filled with 7 μl internal solution containing (in mM): 130 potassium gluconate, 8 KCl, 2 CaCl_2_, 1 MgCl_2_, 10 EGTA, 10 HEPES, 2 Mg-ATP, 0.3 GTP-Na (all from Roth; mOsm = 290-295, pH = 7.3), using 20 μl microloader pipette tips (Eppendorf; cat. no. 5242 956.003). Pipette holders were mounted on and controlled using micromanipulators from Scientifica. Whole-cell patch-clamp recordings were obtained with an EPC10 USB double patch-clamp amplifier from HEKA and the corresponding Patchmaster software. Action potential threshold and peak current was determined by recording currents for 50 ms at 10 mV steps from −60 to 20 mV with 20 μs sampling intervals; between sweeps cells were returned to −60 mV holding potential. The resting membrane potential was determined by the average of three 250 ms recordings in current clamp at 0 pA with 50 μs sampling intervals; spontaneous action potentials that occurred during recordings were excluded from analysis. Recording files were managed using IGOR Pro (Wavemetrics; version 6.22A), and the Patcher's Power Tools extension for HEKA files.

### Imaging of endogenous neuronal activity

Long-term Ca^2+^ imaging of non-stimulated, endogenous neuronal activity was performed in neurons co-transduced with 3 vectors: at 24 h after seeding, cells were co-transduced with AAV-6-Bcl-Xl and AAV-6-GCaMP6f at a titre of 0.3 × 10e8 tu/250.000 neurons each. 48 h later, medium was completely replaced with fresh NeuroBasal, and cells were transduced with vectors expressing either nuclear mCherry (NmC) only, or expressing α-Syn or γ-Syn from one transcription unit, and NmC from a second transcripton unit, at 1 × 10e8 tu/250.000 cells.

Cells were imaged on a Zeiss Observer Z1 microscope equipped with Pecon heating incubator M24 controlled by TempModule S and CO_2_ Module S to maintain them at 37°C and 5% CO_2_. At 10, 15 and 25 days after transduction with synuclein expressing vectors, cells were imaged through a 20x LD PlnD 0.4 objective for 180 seconds in randomly selected areas of the respective well with Zeiss ZEN software. Standard EGFP and DsRed filters were used to record a video for Ca^2+^ transients and an image of NmC fluorescence for segmentation. Segmentation was performed with Fiji (ImageJ) and analysis of coordinated neuronal activity with the FluoroSNNAP software package (Patel et al., 2015).

### Western blot analysis

Protein lysates obtained from cultured neurons were analyzed by denaturing SDS-PAGE (20 μg protein / lane), blotting to nitrocellulose or PVDF membranes and incubation with the following primary antibodies: rat α-Syn, Abcam ab87599 or CST #4179; human α-syn, Invitrogen 32-8100; phospho S129 α-Syn Abcam ab168381; cleaved caspase 3, CST #9661; cleaved caspase 9, CST #9507; phospho-p54/p46 SAPK/JNK, CST #4668; phospho-p38 MAPK, CST #9216; phospho-p44/p42 ERk1/2, CST #4376 or CST#4377; p44/p42 Erk1/2, CST # 9102; Tom20, SantaCruz 11413; VDAC-1, Abcam 15895; LC3B-I / -II, CST #2775; Bim-1, CST # 2933; phospho-AKT, CST #9271; Akt, CST #9272; Bcl-Xl, CST #2764; phospho-Bad (S136), CST #9295; c-Ret (C-20), SantaCruz 1290; TrkA, CST #2508; TrkB, CST #4603; TrkC, CST #3376; EGFP, Roche 11814460001; ß-Tubulin, Sigma, T-4026; Chemoluminescent detection was performed on X-ray film or a BioRad ChemiDoc XRS+ Imager.

### Determination of α-Syn -Bcl-Xl interaction by NMR

Recombinant human α-Syn 1-140 was produced in BL21 *E. coli* and purified as described (Hoyer et al., 2002). The soluble domain (residues 1-209) of human Bcl-Xl was amplified by PCR using primers designed to introduce restriction sites for Nde I and BamH I endonucleases and cloned into the pET15b expression vector. BL21 *E. coli* cells were transformed with the resulting construct, grown at 37°C and protein synthesis was induced overnight at 20°C with 0.5 mM IPTG (isopropyl beta-D-1 thiogalactopyranoside). Under these conditions of subcloning, the protein is expressed with a N-terminal 6XHis tag followed by a thrombin digestion site. After induction, the bacterial cells were sonicated, centrifuged 15 min at 15000 g, and the clarified supernatant was loaded onto a 5 ml nickel-sepharose column equilibrated with 20 mM Tris-HCl pH 7.5, 0.5 M NaCl, 10 mM imidazole. The resin was washed with the same buffer containing 50 mM imidazole (10 column volumes) and the protein was eluted with 2 column volumes of buffer with 250 mM imidazole. After dialysis, the tag was removed by thrombin digestion and the protein was further purified by reverse immobilized-metal affinity chromatography (IMAC) and size exclusion chromatography in a Superdex G200 column. Complete removal of the tag was assessed by Western blot analysis using an anti-His-HRP-conjugated antibody. After cleavage of the 6XHis tag, the amino acids GSH remain at the Bcl-Xl N-terminus.

For the production of ^15^N-labeled proteins, host cells were grown in M9 minimal medium using ^15^NH_4_Cl (Cambridge Isotope Laboratories) as sole nitrogen source.

α-Syn backbone resonance assignment was available from previous studies (Bertoncini et al., 2005). For Bcl-Xl assignment, we used the chemical shift deposited under BMRB code: 19521 (Follis et al., 2014).

α-Syn (1-26) peptides were produced with and without an acetyl- group at the N-terminus, by standard Fmoc-solid-phase peptide synthesis using an ABI 433A synthesizer (Applied Biosystems). Peptides were purified by reversed-phase HPLC and the pure product verified by MS, lyophilized and then extensively dialyzed.

^1^H-^15^N HSQC spectra were recorded at 298K on 600 MHz and 700 MHz Bruker spectrometers, equipped with cryogenic triple-resonance probe. Proteins and peptides were measured in Hepes 25 mM pH 7.0, NaCl 50 mM, 10% D_2_O, 1mM DTT.

The combined ^1^H and ^15^N chemical shift perturbation (csp) values were determined with the following equation: csp = [(ΔδH_N_)^2^+(ΔδN/5)^2^]^1/2^, where ΔδH_N_ and ΔδN are the chemical shift changes measured in the ^1^H and ^15^N frequency dimensions, respectively. Titration curves, with ΔδN chemical shift changes plotted vs. ligand concentrations, were fitted by a single binding model to obtain the dissociation constant of the interaction (Kd) (Delaglio et al., 1995). NMR data were processed and analyzed using NMRPipe (Delaglio et al., 1995) and Sparky (T. D. Goddard and D. G. Kneller, University of California, San Francisco).

### Statistical analysis

Experimental data were analyzed for statistical significant differences between groups by 1-way ANOVA with Tukey's *post hoc* test. Statistical powers of all such comparisons were analyzed by G^*^Power3.1 (Faul et al., 2009) with the following settings: test family = *t*-tests (2-tailed); statistical tests = difference of means between 2 independent groups; type of power analysis = *post hoc*; effect size *d* computed from means and standard deviations of groups to compare; α error probability = 0.05; with given sample sizes the power of the statistical assessment was computed as (1–ß error probability). A reasonable statistical power of the respective statistical analysis was assumed at 1–ß error probability > 0.8.

## Results

All experiments of this study were performed in primary cortical neurons obtained from E18 rats, seeded at 250,000 cells per well in 24-well plates. After 3 days of culture the cells were transduced with AAV-6 vectors expressing the respective synuclein [human α-Syn, β-Syn or γ-Syn, occasionally plus a fluorophore (cytoplasmic EGFP or nucleus-targeted mCherry) from an independent expression cassette (Supplemental Figure 1)], or only EGFP. Viral titres were adjusted to express the human synucleins in a 15–20-fold excess over the endogenous rat α-Syn, as this was the lowest dose of human α-Syn to cause a slowly progressive but robust neurodegeneration (Taschenberger et al., 2013; and see **Figure 5B**).

In addition, we used AAV-6 vectors expressing fluorescent genetically encoded sensors enabling detection of Ca^2+^ transients, ATP and ROS levels in living neurons. These investigations were mostly performed at 6 dpt (days post transduction; corresponding to div (days *in vitro*) 9) and at 13 dpt (corresponding to div 16). At 6 dpt neurodegeneration is barely evident in terms of loss of neurons, while at 13 dpt a significant number of neurons are already degenerated due to α-Syn's neurotoxicity. At about 18-21 dpt the majority of neurons has degenerated in α-Syn expressing cultures, while almost no neuron loss occurred in γ-Syn expressing cultures.

### Cytoplasmic and mitochondrial calcium handling

Disturbed Ca^2+^ homeostasis and neurodegeneration are intimately linked (Mattson, 2007; Cali et al., 2014). Many recent reports have demonstrated that α-Syn interacts with biological membranes in a way that ion conductive pores are produced (Lashuel et al., 2002; Kagan, 2012). These studies mostly exploited cell free systems or addition of various pre-formed synuclein species to cell cultures (Pacheco et al., 2015; Angelova et al., 2016). Surprisingly few studies have addressed the question if continuous α-Syn overexpression within cells would also result in detectable disturbances of ion fluxes. It was found that in SH-SY5Y cells α-Syn expression led to increased potassium-mediated influx of Ca^2+^ over the cell membrane (Hettiarachchi et al., 2009). Thus, we first asked the question if in excitable cells like neurons, which crucially depend on appropriate ion homeostasis, α-Syn overexpression might result in impaired Ca^2+^ homeostasis in cytosol and/or in mitochondria. Determination of Ca^2+^ dynamics in mitochondria was considered to be of special importance, as these organelles are morphologically severely condensed by α-Syn and ß-Syn overexpression, which is the earliest detectable lesion caused by synuclein overexpression (Taschenberger et al., 2013; and see **Figure 5D**). Mitochondria are proposed to be important targets for α-Syn toxicity due to high cardiolipin content (Ghio et al., 2016), which seems to act as an anchor for synucleins within membranes.

**Figure 1:**
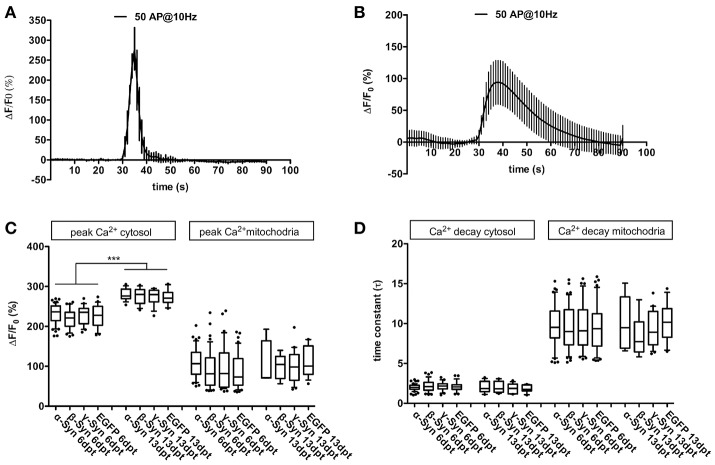
Cytosolic and mitochondrial Ca^2+^ handling under α-Syn overexpression. Representative traces of Ca^2+^ transients obtained after field stimulation (50 AP @ 10 Hz) in cytoplasm **(A)** and in mitochondria **(B)**. Quantification of peak Ca^2+^ influx into cytoplasm and mitochondria **(C)** and quantification of Ca^2+^ decay times from cytoplasm and mitochondria **(D)** is shown for neurons overexpressing α-, β-, γ-Syn, or EGFP for 6 or 13 days, demonstrating that Ca^2+^ transients and buffering are not affected by α-Syn overexpression. Traces show mean ± SD; boxplots show median, 25–75% quartile, whiskers for 10–90% quartile and values outside 10–90% quartile as individual dots; *N* = 28–51 neurons for dpt6 and 12–15 neurons for dpt 13 from at least three independently prepared cultures each per condition in **(C,D)**. Statistical significances were calculated with 1-way ANOVA with Tukey's multiple comparison test. ^***^p < 0.001; statistical power (with G*Power 3.1, two-tailed t-tests for all group-wise comparisons) for observed differences > 0.9 for all groups.

We evaluated the peak level of Ca^2+^ influx into cytoplasm (i.e., Ca^2+^ fluxes over the cell membrane) or mitochondria (i.e., Ca^2+^ fluxes over the mitochondrial membranes) and the decay kinetics of Ca^2+^ clearance from cytoplasm or mitochondria. This was achieved by means of the genetically encoded calcium sensor RCaMP1e, that was targeted to either cytosol or mitochondria (Akerboom et al., 2013), and was co-expressed with α-Syn, ß-Syn, γ-Syn, or EGFP within the neurons. Our results demonstrate that robust and sustained overexpression of α-Syn does not result in detectable disturbances on Ca^2+^ influx or decay rate in cytoplasm or mitochondria of neurons (Figure 1), suggesting that basic ion handling capabilities are not affected by any of the tested synucleins, in contrast to results obtained earlier in SH-SY5H cells (Hettiarachchi et al., 2009). Results shown in Figure 1 were obtained by field stimulation (FS), very similar data were obtained by K^+^–mediated depolarization (not shown). Using fewer action potentials (APs) for field stimulation resulted in proportionally smaller transients without changing influx or efflux kinetics significantly (not shown).

In older neurons, i.e., at 16 div, corresponding to 13 dpt, peak Ca^2+^ influx into cytosol after field stimulation was significantly higher than in younger neurons (div 9, dpt 6), but no differences between α-Syn and controls were detected (Figure 1C). Resting Ca^2+^ levels (i.e., in absence of any stimulus; measured with the ratiometric Ca^2+^ sensor D3cpV) were found to be indistinguishable between all conditions (not shown). Thus, in primary neurons, α-Syn does not seem to impair Ca^2+^ handling capabilities.

### Neuronal excitability and basic electrophysiological properties

**Figure 2:**
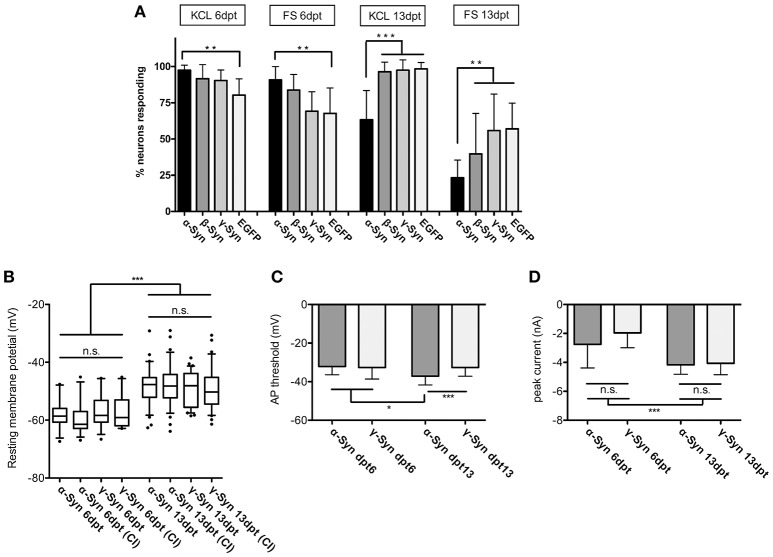
Excitability and membrane properties of neurons under α-Syn overexpression. **(A)** Imaging of RCaMP1e fluorescence shows that the fraction of neurons responding to K^+^-mediated or electrical stimulation is higher at 6 dpt and lower at 13 dpt in α-Syn overexpressing cells as compared to controls. *N* = 8 cultures with 40–50 neurons within the visual field each. **(B)** Electrophysiological determination of the resting membrane potential using patch-clamp reveals no differences between α-Syn and γ-Syn expressing neurons in resting states or after current injections (CI). **(C)** Determination of the threshold potential at which neurons start to evoke action potentials shows that at 13 dpt α-Syn overexpressing neurons start firing at less depolarization. **(D)** Peak currents evoked by spiking are not significantly different between α-Syn and γ-Syn expressing neurons. Boxplot shows median, 25–75% quartile, whiskers for 10–90% quartile and values outside 10–90% quartile as individual dots; bars show mean ± SD. *N* = 14–15 neurons for dpt6 and 30–34 neurons for dpt13 in **(B–D)**. Statistical significances were calculated with 1-way ANOVA with Tukey's multiple comparison test. n.s, not significant; ^*^p < 0.05; ^*^p < 0.01; ^***^p < 0.001. Statistical powers: in **(A)**: ^**^KCl dpt 6 > 0.95; ^**^FS dpt 6 > 0.85; ^***^KCl dpt13 > 0,95; ^**^FS dpt13 > 0,95; in **(B)**: ^***^ > 0.95; in **(C)**: ^***^ = 0.75; ^*^ = 0.46; D: ^***^ > 0,95.

At 6 dpt the fraction of neurons responding to K^+^ or FS (i.e., those neurons which showed a ΔF/F0 above the background fluorescence of RCaMP1e) was similar in neurons overexpressing α-Syn, ß-Syn, γ-Syn, or EGFP. At this time, we found a moderate but significantly higher number of α-Syn overexpressing neurons to respond to the stimulus, as compared to EGFP expressing neurons. However, longer-term α-Syn overexpression (at 13 dpt) resulted in a significant decrease in the number of neurons responding to K^+^- mediated or electrical stimulation (Figure 2A). Here, K^+^–mediated depolarization [ = opening of voltage-dependent Ca^2+^ channels (VDCCs)] resulted in Ca^2+^ influx and thus increased sensor fluorescence in only 63 ± 20% of RCaMP1e expressing neurons, as compared to about 96% in β-Syn-, γ-Syn- or EGFP expressing neurons. A similar relation was obtained by electrical field stimulation, resulting in Ca^2+^ influx and increased sensor fluorescence in 23 ± 12 % of α-Syn expressing neurons, as compared to 40 ± 27% in ß-Syn expressing neurons, 56 ± 25% in γ-Syn expressing neurons and 57 ± 17% in EGFP expressing neurons.

These results demonstrate that neuronal reaction to external stimuli is reduced after long-lasting α-Syn overexpression, and thus α-Syn may cause disturbances of neuronal communication capabilities. This effect was not detected for ß-Syn after K^+^ stimulation, while a trend toward reduced excitability was found for ß-Syn overexpressing neurons after FS, which, however, did not reach statistical significance under our experimental conditions.

In order to determine if this diminished reaction to external stimuli is correlated with basic membrane properties of α-Syn overexpressing neurons, we subjected α- and γ-Syn overexpressing neurons to patch-clamp experiments. These studies showed that resting membrane potentials were significantly different between neurons assessed at 6 dpt or 13 dpt, but were not different between α-Syn and γ-Syn overexpressing neurons at any time point (Figure 2B). Next we determined the depolarization threshold necessary to evoke action potentials (Figure 2C). At 6 dpt no differences between α- and γ-Syn overexpressing neurons were detectable. Somewhat counterintuitive to results obtained by K^+^- or field stimulation, α-Syn overexpressing neurons started to fire action potentials at significantly less depolarization level at 13 dpt (α-Syn: −37 ± 3 mV vs. γ-Syn:−32+/−4 mV). However, the peak current influx through voltage gated channels under these conditions was identical between α- and γ-Syn overexpressing neurons at both 6 and 13 dpt, although neurons at 13 dpt showed a more pronounced peak current influx than neurons at 6 dpt (Figure 2D). Thus, while α-Syn diminished neuronal responses to external stimuli, basic electrophysiological membrane properties appeared not to be altered by α-Syn overexpression.

### Mitochondrial energy production by oxidative phosphorylation

Limited neuronal reaction to external stimuli could result from impaired energy production. The electron transport chain of mitochondria is proposed to be an important target of α-Syn mediated neurotoxicity, and toxins inhibiting complex I are proven tools to induce PD-like symptoms in experimental animal models (Zhu and Chu, 2010). α-Syn has been reported to interact directly with mitochondria(Gautier et al., 2014; Ryan et al., 2015; Zaltieri et al., 2015), especially by cardiolipin-mediated membrane binding (Ghio et al., 2016). Within the mitochondrial membrane(s), α-Syn could potentially impact on membrane tension and curvature (West et al., 2016), thereby inflicting upon essential mitochondrial functions like oxidative phosphorylation (OXPHOS) through the membrane-bound respiratory chain. We investigated this potential impact of α-Syn on mitochondrial functionality by exploiting the genetically encoded fluorescent ATP sensor Ateam 1.03 to record both ATP production and consumption rates. This sensor was recently shown to be able to quantitatively report cytoplasmic ATP levels in primary neurons (Toloe et al., 2014). It offers the added advantage of individual neuron imaging over measurements of oxygen consumption, which cannot discriminate between α-Syn expressing neurons and the non-affected astrocytes in the culture.

**Figure 3:**
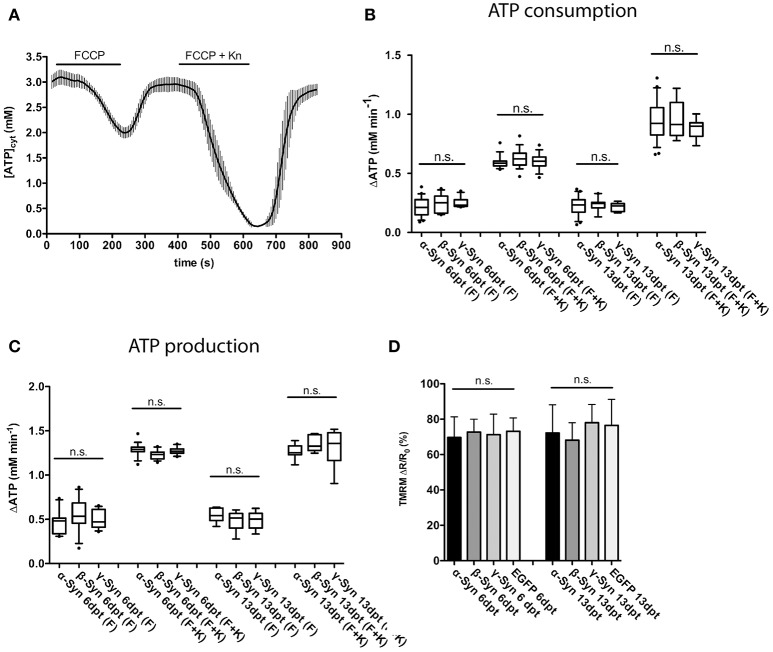
Mitochondrial ATP production and membrane potential are not affected by α-Syn overexpression. FCCP-mediated uncoupling and subsequent recording of ATP consumption and production rates assayed the overall integrity of the mitochondrial respiratory chain. A typical trace of cytosolic ATP levels as measured with the Ateam1.03 fluorescent sensor after FCCP uncoupling, washout, a second uncoupling by FCCP plus kainate intoxication and again washout is shown in **(A)**. The rates of cytoplasmic ATP consumption after FCCP (F) or FCCP+kainate (F+K) in neurons expressing α-, β-, or γ-Syn for 6 or 13 days is shown in **(B)**. The rates of mitochondrial ATP production after washout of FCCP (F) or FCCP+kainate (F+K) in neurons expressing α-, β-, or γ-Syn for 6 or 13 days is shown in **(C)**. Mitochondrial membrane potential assessed with the dye TMRM in neurons expressing α-, β-, γ-Syn or EGFP for 6 or 13 days is shown in **(D)**. Traces show mean ± SD, boxplots show median, 25–75% quartile, whiskers for 10–90% quartile and values outside 10–90% quartile as individual dots. *N* = 21–28 neurons per condition from at least three independent cultures. Statistical power for not missing any significant differences at *p* < 0.05 is > 0.85 for all groups.

In order to determine the ability of mitochondria to synthesize ATP in the presence of α-Syn levels sufficient to induce neurodegeneration (see **Figure 5** for details), the following protocol was used: at 6 or 13 days after AAV-Synuclein transduction, mitochondria were uncoupled with FCCP to inhibit ATP production and the rate of ATP decline (i.e., ATP consumption) in cytoplasm was measured; after washing out FCCP, the proton gradient is re-established through activity of the respiratory chain, mitochondrial ATP is re-produced and the rate of ATP production was determined. In order to demonstrate that this FCCP application has not terminally damaged mitochondria, a second intoxication with FCCP plus kainate was performed, resulting in even more pronounced depletion and subsequent re-production of ATP, suggesting that during the first FCCP application all data recorded by the sensor where within its dynamic range. A typical trace of such a recording is shown in Figure 3A.

Our data show that both, ATP consumption after mitochondrial uncoupling (Figure 3B) and, more importantly, ATP re-production after FCCP wash-out (Figure 3C) were not negatively affected by α-Syn, as compared to the γ-Syn control, despite the substantial structural deformation caused by α-Syn in mitochondria (see **Figure 5D**). ß-Syn also had no effect on ATP consumption or re-production. These data suggest that α-Syn overexpression does not directly impair oxidative phosphorylation and thus the neuron's energy production capabilities. We furthermore measured the mitochondrial membrane potential with the fluorescent dye TMRM and again could not detect any difference between α-Syn and γ-Syn controls (Figure 3D). Thus, while α-Syn causes morphological alterations in neuronal mitochondria, it does not concomitantly impair their capability to synthesize ATP.

### Generation of reactive oxygen species

In an attempt to reinforce our investigation on mitochondrial impairments that might be caused by α-Syn overexpression, we quantified cytoplasmic and mitochondrial ROS production with the ROS sensor roGFP1 (Cannon and Remington, 2008). In principle, ROS are normal by-products of electron transport through the respiratory chain, since inevitably a certain percentage of electrons escape from it, leading to formation of ${\text{O}}_{2}^{.-}$ and subsequently H_2_O_2_. Increased ROS levels can have several reasons, but generally indicate that the respiratory chain performs sub-optimally.

**Figure 4:**
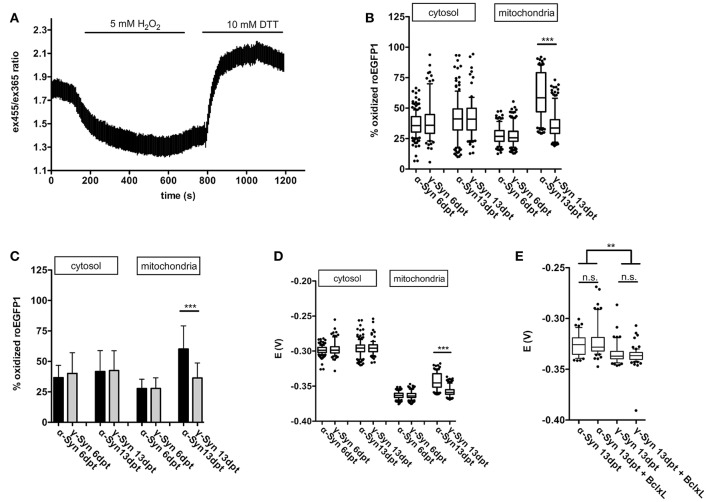
Mitochondrial ROS production is increased by α-Syn overexpression. A typical calibration trace for roGFP1 expressed in cytoplasm is shown in **(A)**. The percentage of roGFP1 molecules in oxidized state for cytoplasmic or mitochondrial expression, either at 6 or 13 days after α-Syn or γ-Syn overexpression is shown as a boxplot in **(B)**, the same data are shown as bars ± SD in **(C)** for the sake of clarity. The redox potential of roGFP1 calculated for cytosol or mitochondria either at 6 or 13 days after α-Syn or γ-Syn overexpression is shown in **(D)**. The redox potential from an independent series of experiments including co-expression of Bcl-Xl is shown in **(E)**. *N* = 125–150 neurons per condition. Traces show mean ± SD, boxplots show median, 25–75% quartile, whiskers for 10–90% quartile and values outside 10–90% quartile as individual dots, bars show mean ± SD. Statistical analysis by 1-way ANOVA and Tukey's *post hoc* test. ^***^p < 0.001; ^**^p < 0.01; n.s. not significant. Statistical powers > 0.95 for comparisons between all groups.

Cytoplasmic expression of roGFP1 did not reveal any changes in redox potential induced by α-Syn overexpression as compared to γ-Syn overexpressing controls. The cytoplasmic roGFP1 sensor (calibration curve shown in Figure 4A) was used earlier in quantification of changes in cytoplasmic redox state caused by MeCP2 overexpression, and thus has the proven capability to detect even relatively subtle differences in redox potential (Grosser et al., 2012). In contrast, targeting expression of roGFP1 to mitochondrial matrix showed that the mitochondrial redox potential was significantly shifted toward a more oxidized state through α-Syn overexpression. This effect was detectable at 13 dpt but not at 6 dpt and was absent after γ-Syn expression. (Figures 4B–D). Co-expression of Bcl-Xl, which is capable of preventing α-Syn-induced neurodegeneration in this model (see below), did not prevent the increase in oxidized mitochondrial milieu induced by α-Syn (Figure 4E). It should be noted that absolute values for the mitochondrial redox potential shown in Figures 4D,E are from independent experimental series. Ro1GFP reports significantly different absolute mitochondrial redox potentials between experiments, suggesting that experimental conditions might have influence on this parameter. Thus, absolute values for mitochondrial redox potential delivered by ro1GFP might not be as reliable as the difference detected between α-Syn and γ-Syn expressing neurons, which was found to be conserved between experiments.

Thus, on the one hand our data clearly demonstrate the capability of the respiratory chain to adequately respond to even substantial energy demands in the presence of robust levels of α-Syn overexpression. On the other hand, however, α-Syn appears to increase the loss of electrons while transported through the respiratory chain, resulting in a more oxidizing state within the mitochondrial matrix. This effect appears to depend on longer-term presence of α-Syn in the neurons, as it is evident only at 13 days of overexpression, but not at 6 days of overexpression. During the time course of these experiments this increase in ROS formation in mitochondria was not sufficient to impact on the proton motion force driving ATP production, but in the long run may cause functional impairments of essential mitochondrial components i.e., by protein, lipid or DNA oxidation, by initiating remote actions through serving as signaling molecules, or by inflicting on integrity of the mitochondrial permeability transition pore (mPTP) complex.

### Protection from synuclein toxicity

The increased mitochondrial ROS production and more oxidized redox potential might serve as trigger to induce neuronal degeneration after about 10-12 days of α-Syn overexpression. However, the impact of increased ROS could be considered moderate on first sight, as exemplified by the ability of affected mitochondria to still produce ATP as demanded. As such, the question remains how α-Syn, and to lesser extent β-Syn, eventually induce neuronal death in this paradigm.

**Figure 5:**
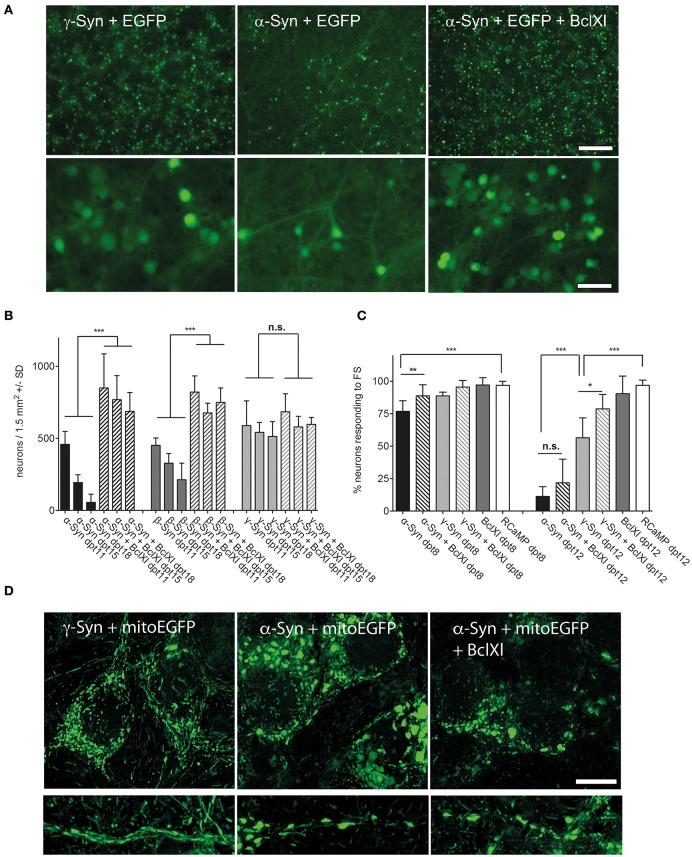
Bcl-Xl prevents synuclein induced neuronal loss, but not α-Syn-induced loss of excitability and mitochondrial deformation. **(A)** Low magnification (upper panel) and high magnification (lower panel) photomicrographs of neurons expressing γ-Syn and EGFP (left), α-Syn and EGFP (middle) or α-Syn and EGFP and Bcl-Xl (right) at 15 days after transduction with the respective AAV vectors (synuclein/EGFP = 1 × 10e8 tu; Bcl-Xl = 3 × 10e7 tu). Scale bar = 400 μm in upper panel and 100 μm in lower panel. **(B)** Numbers of neurons counted at days 11, 15, and 18 after transduction with the respective AAV vectors demonstrating almost complete prevention of α-Syn-induced neuron loss by Bcl-Xl. Statistical analysis by 1-way ANOVA with Tukey's *post hoc* test. Statistical power for significantly different values in all groups > 0.95. **(C)** Percentage of neurons that are excitable by field stimulation at 8 or 12 days after transduction with the respective AAV vectors (α-Syn/EGFP + RCaMP1e (black bars); α-Syn/EGFP + Bcl-Xl + RCaMP1e (hatched black bars)); γ-Syn/EGFP + RCaMP1e (light gray bars); γ-Syn/EGFP + Bcl-Xl + RCaMP1e (hatched light gray bars); Bcl-Xl + RCaMP1e (gray bars); or RCaMP alone (white bars). Differences in total vector titre were adjusted with an AAV expressing no transgene. Statistical power > 0.8 for significantly different values at 8 dpt, and > 0.9 for statistically significant values in all groups at dpt12; except for the difference between γ-Syn and γ-Syn + Bcl-Xl at dpt12, where statistical power is 0.76. **(D)** Representative micrographs (maximum projection of deconvoluted Z-stacks) of mitochondrial morphology in soma (upper panels) and proximal neurites (lower panels) of neurons overexpressing γ-Syn (left panels), α-Syn (middle panels) or α-Syn + Bcl-Xl (right panels) at 13 dpt, demonstrating that Bcl-Xl does not rescue the α-Syn-induced deformed phenotype of mitochondria. ^***^p < 0.001; ^**^p < 0.01; ^*^p < 0.05; n.s. not significant. Scale bar = 10 μm.

This issue was addressed by two complementary approaches. On the one hand, we analyzed protein lysates of α-, β-, and γ-Syn expressing neurons for canonical stress kinase signaling, which might be triggered by factors not picked up by the fluorescent sensors (see below). On the other hand we analyzed several potentially neuroprotective factors for their capability to counteract synuclein's neurotoxicity. Although somewhat indirect, analysis of pathways protecting neurons from α-Syn overexpression might shed light on mechanisms that lead to neuronal degeneration. This latter approach revealed that neurotrophins BDNF, CDNF, or GDNF, either expressed from AAV vectors or added as recombinant peptides, could not ameliorate the degeneration of α-Syn or β-Syn overexpressing neurons (Supplemental Figure 2). Furthermore, expression of Calpastatin, a highly effective inhibitor of the neurodegeneration-inducing calpain proteases (Simões et al., 2012) and CathepsinD, a protease proposed to act as a α-Syn degrading factor (Cullen et al., 2009; Shevtsova et al., 2010) did not show any neuroprotection in our paradigm (Supplemental Figure 3). The only protein tested so far that indeed almost completely prevented α- and β-Syn mediated neurodegeneration was Bcl-Xl, an anti-apoptotic factor known to prevent permeabilization of the outer mitochondrial membrane (OMM) (Boise et al., 1993; Zheng et al., 2016) (Figure 5). This neuroprotective effect of Bcl-Xl appeared to be dosage dependent (Supplemental Figure 4A). Importantly, Bcl-Xl prevented loss of α-Syn overexpressing neurons not only for the wildtype α-Syn protein, but also for the genetic mutants A30P and A53T. Furthermore, Bcl-Xl protected neurons from overexpression of the artificial mutant TP (triple proline = A30P, A56P, A76P), which was designed to generate increased levels of oligomeric α-Syn species and has reduced propensity to fibrillate (Karpinar et al., 2009).

**Figure 6:**
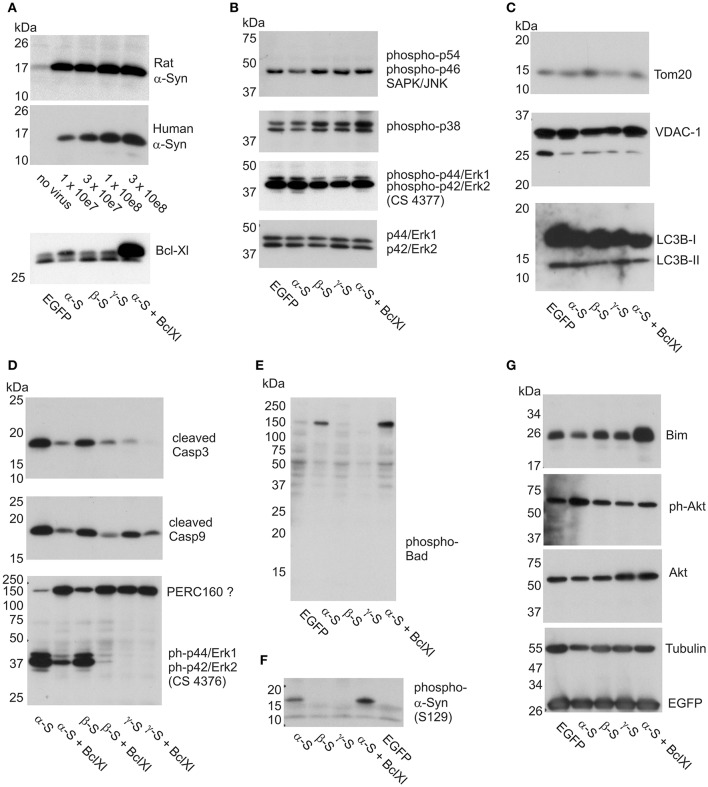
Impact of α-Syn overexpression on stress kinases, caspases, and apoptosis-related proteins. Western blots are shown for cell lysates isolated from cortical neurons at 12 days of synuclein overexpression under denaturing conditions and electrophoresed on SDS-PAGE. **(A)** Expression analysis of AAV-expressed rat and human α-Syn at different AAV titres and Bcl-Xl (at 3x10e7 tu) to delineate the level of overexpression (see Materials and Methods for more details). **(B)** Detection of levels of phosphorylated JNK (panel 1), phosphorylated p38 (panel 2) and phosphorylated Erk (panel 3; antibody CS4377) stress kinases, detection of non-phosphorylated Erk is shown in panel 4 as loading control. **(C)** Detection of the levels of mitochondrial proteins Tom-20 (panel 1) and VDAC-1 (panel 2), and lysosomal proteins LC3B1 and −2 (panel 3). **(D)** Detection of levels of cleaved caspase 3 (panel 1), cleaved caspase 9 (panel 2), and phospho-Erk (panel 3; antibody CS4376). **(E)** Detection of phospho-Bad in a high molecular weight complex. **(F)** Detection of phosphorylated α-Syn (S129). **(G)** Detection of Bim and Akt, Tubulin and EGFP (which is co-expressed with the synucleins) as loading controls. EGFP, neurons transduced with AAV-EGFP (1 × 10e8 tu); α-S, neurons transduced with AAV-α-Syn-EGFP (expressing α-Syn and EGFP from independent transcription units, 1 × 10e8 tu). ßS, neurons transduced with AAV-ß-Syn-EGFP (1 × 10e8 tu); γ-S, neurons transduced with AAV-γ-Syn-EGFP(1 × 10e8 tu); Bcl-Xl, neurons transduced additionally with AAV-Bcl-Xl (3 × 10e7 tu).

These results clearly indicated that α-Syn and β-Syn overexpression both trigger cell death processes closely related to mitochondria outer membrane permeabilization, as this is Bcl-Xl's major site of action. This assumption was then proven by detection of activated caspases in later stages of α-Syn expression, and prevention of this activation by Bcl-Xl (Figure 6D). It should be noted that we did not find “upstream” apoptotic events like translocation of annexin V—detectable phosphatidylserine to the cell membrane (not shown).

However, while prevention of synuclein-induced neuronal cell loss was impressive, the reduction in relative numbers of excitable neurons by long-term α-Syn overexpression could be prevented by Bcl-Xl only at 8 dpt, but not at 12 dpt (Figure 5C). Furthermore, the spherical phenotype of mitochondria that is caused by α-Syn overexpression cannot be rescued by Bcl-Xl (Figure 5D). None the less, the fact that Bcl-Xl protects from α-Syn's neurotoxicity strongly argues that α-Syn causes neuron death by interaction with the OMM, either directly or mediated by oxidized mitochondrial thiol components.

### α-Syn and Bcl-Xl interaction

The neurons used in this study contain endogenous levels of Bcl-Xl (Figure 6A), which might be sufficient to protect them from low-level α-Syn overexpression. Increasing the expression level of Bcl-Xl results in increased protection from higher level α-Syn overexpression (Supplemental Figure 4A). This relation could be indicative for a direct interaction of α-Syn and Bcl-Xl, where increased levels of Bcl-Xl would sequester α-Syn away from mitochondria or from mitochondrial sites were α-Syn induces pathological processes. NMR studies indeed revealed a transient interaction between α-Syn and Bcl-Xl. The binding mainly involved the first 10 N-terminal residues of α-Syn (Supplemental Figure 5) and residues within the C-terminal region of Bcl-Xl (Supplemental Figure 6). Interaction studies performed with synthetic peptides showed that N-terminal acetylation of synuclein induced larger chemical shifts changes for many of the residues perturbed, suggesting a more efficient binding. The binding affinity of the interaction was determined with the help of NMR spectroscopy using ^15^N-Bcl-Xl and N-terminal acetylated alpha-synuclein peptide 1-26. The averaged Kd, determined based on titration experiments and fitting analysis of residues 94, 120, 134 and 196 of Bcl-Xl, was estimated as 1.95 ± 0.56 mM, indicating that α-Syn can bind to Bcl-Xl although with only weak affinity. It should be noted that the k_d_ for binding of pro- and anti-apoptotic proteins to each other is below 10 nM, and thus shows 2 orders of magnitude higher affinity than that of α-Syn and Bcl-Xl binding. These results suggest that Bcl-Xl protects from α-Syn's neurotoxicity most likely not by sequestration of α-Syn away from mitochondria but by a mitochondria-specific rescue mechanism like prevention of damage of the OMM.

### Kinase signaling and apoptosis

With respect to putative α-Syn-induced signaling we investigated cell lysates for activation of three major kinase pathways, converging on SAPK/Jun, p38 MAPK, or Erk1/2 kinases. While SAPK/Jnk kinase and p38 MAPK are heavily involved in apoptotic signaling events, and are thus the canonical stress kinases, ERK 1/2 is activated rather by differentiation and plasticity-promoting factors and exerts pro-survival signaling events (Harper and Wilkie, 2003). However, a role in neurodegeneration has also been claimed for this type of kinase (Subramaniam and Unsicker, 2010), and, more important in the context of this study, all MAPK can be activated following exposure to ROS (Lin et al., 2016). Thus, their activation would also serve as a surrogate marker for ROS that might have escaped from mitochondria but may not be detected by the cytoplasmic ro1GFP sensor.

We found that levels of phosphorylated, i.e., activated, forms of these kinases were very similar irrespective if either no synuclein, or α-, β-, or γ-Syn was overexpressed. Figure 6B shows levels of phospho-p54/p42 SAPK/Jnk (first panel), phospho-p38 MAPK (second panel), phospho-Erk1/2 (third panel) and as controls levels of non-phoshorylated Erk1/2 as loading controls, all detected at 12 days of synuclein overexpression. Identical relative levels of these kinases were detected at 6 days of synuclein overexpression (not shown). Thus, synuclein expression does not seem to activate or silence canonical stress kinase signaling in neurons.

We also found that despite morphological deterioration of mitochondria through α-Syn overexpression no loss of mitochondrial proteins Tom20 and VDAC-1 was detectable (Figure 6C, panels 1 and 2). Furthermore, conversion of LC3B-I to LC3B-II was absent in these neurons at 12 days of synuclein overexpression, indication no contribution of autophagy to neuronal degeneration (Figure 6C, panel 3). However, congruent with the neuroprotective effects found for Bcl-Xl, we detected robust activation of caspase 3 through α-Syn and β-Syn overexpression, which was largely prevented by Bcl-Xl co-expression (Figure 6D, panel 1). Activation of caspase 9 was also evident, however, cleaved (activated) caspase 9 was also detected in cultures overexpressing γ-Syn (Figure 6D, middle panel) and might rather reflect the basic turnover of neurons or glia. A more specific marker for neurodegeneration induced by α-Syn and β-Syn was found by exploiting a different phospho-Erk1/2 antibody than the one used to quantify Erk1/2 activation state in Figure 6B. While phospho-Erk antibody CS4377 detects phosphorylation at Tyr204 of Erk1 (= Tyr187 in Erk 2) (Figure 6B, panel 3), antibody CS4376 detects phosphorylation at Thr202 of Erk1 (= Thr187 in Erk2) (Figure 6D, panel 3).

With the latter antibody, we detected a signal for phospho-Erk1/2 not at the predicted molecular weight of 42/44 kDa, but predominately at a larger size of apparent MW of 160 kDa. A signaling complex of this size containing Erk1/2 has been described in neurons earlier, termed PERC-160 (Lundquist and Dudek, 2006).

Intriguingly, α-Syn and β-Syn expression appeared to cause breakdown of this complex or separation of Erk1/2 from it, as expression of both synucleins result in largely diminished signal at 160 kDa and appearance of a strong signal at the predicted MW at 42/44 kDa. This appearance of Thr202 phosphorylated Erk1 / Thr187 phosphorylated Erk2 was not detected in γ-Syn expressing control neurons. As shown in Supplemental Figure 7, this apparent complex breakdown becomes detectable only at relatively long-term of α-Syn and β-Syn overexpression, and thus cannot serve as a predictive early marker of synuclein toxicity. Its prevention by Bcl-Xl co-expression suggests that this phenomenon is a consequence rather than a cause of α-Syn / β-Syn induced apoptosis.

A signal very specific for α-Syn overexpression was unexpectedly found in the pro-apoptotic BH3 domain-only protein Bad, which became phosphorylated at serine 136 specifically upon α-Syn overexpression (Figure 6E). Interestingly, phospho-Bad was not detected at its monomeric molecular weight of 23 kDa but only within a 160 kDa complex. This result appeared to be counterintuitive insofar, as Bad phosphorylation is considered to be a pro-survival and anti-apoptotic signal in neurons (Datta et al., 1997; Roy et al., 2009). Thus, for the time being, it remains elusive if Bad phosphorylation upon α-Syn overexpression has functional consequences. Notably, Bcl-Xl did not interfere with phosphorylation of Bad, or with phosphorylation of α-Syn at S-129 (Figure 6F). An unexpected finding of Bcl-Xl co-expression with α-Syn was the evident up-regulation of Bim (Figure 6G, first panel), which, as Bad, belongs to the BH3-domain only class of pro-apoptotic proteins. Bim induces apoptosis by binding to Bcl2-or Bcl-Xl and thus antagonizing their anti-apoptotic function. However, Bim was not up-regulated through α-Syn in our paradigm. Furthermore, we found no decline in pro-survival triggers like Akt through α-Syn overexpression (Figure 6G, second and third panel).

A functional consequence of α-Syn overexpression important for maintenance of dopaminergic neurons is downregulation of the receptor tyrosine kinase c-Ret, which serves as the co-receptor for neurotrophic factor GDNF (Decressac et al., 2012). Ret is expressed in cortical neurons predominately as a truncated 60 kDa form, and only to a minor part as the full lenght 150 kDa receptor. α-Syn and to lesser extent β-Syn, but not γ-Syn, cause robust depletion of both full length and truncated c-Ret (Supplemental Figure 8A), but not of neurotrophin receptors trkA, trkB, or trkC (Supplemental Figure 8B), implicating that cultured cortical neurons recapitulate α-Syn induced lesions found in dopaminergic neurons *in vivo*.

### Endogenous synchronized network activity of α-Syn overexpressing neurons

Bcl-Xl protects against the neurotoxicity of α-Syn in a way that mitochondrial outer membrane perforation (MOMP) and resulting caspase activation is prevented and thus cells do not undergo apoptotic degeneration. However, structural mitochondrial deformation, increased mitochondrial ROS formation and diminished reaction to external electrical stimulation persist. This finding opened up novel research opportunities. We could now study long-term effects of α-Syn overexpression that otherwise could not be addressed in cultured neurons, at least not at α-Syn expression levels that have a proven impact on neuronal health. By exploiting Bcl-Xl mediated prevention of apoptosis we analyzed if α-Syn would have an impact on the endogenous synchronized network activity of cultured cortical neurons, especially in aged cultures.

**Figure 7:**
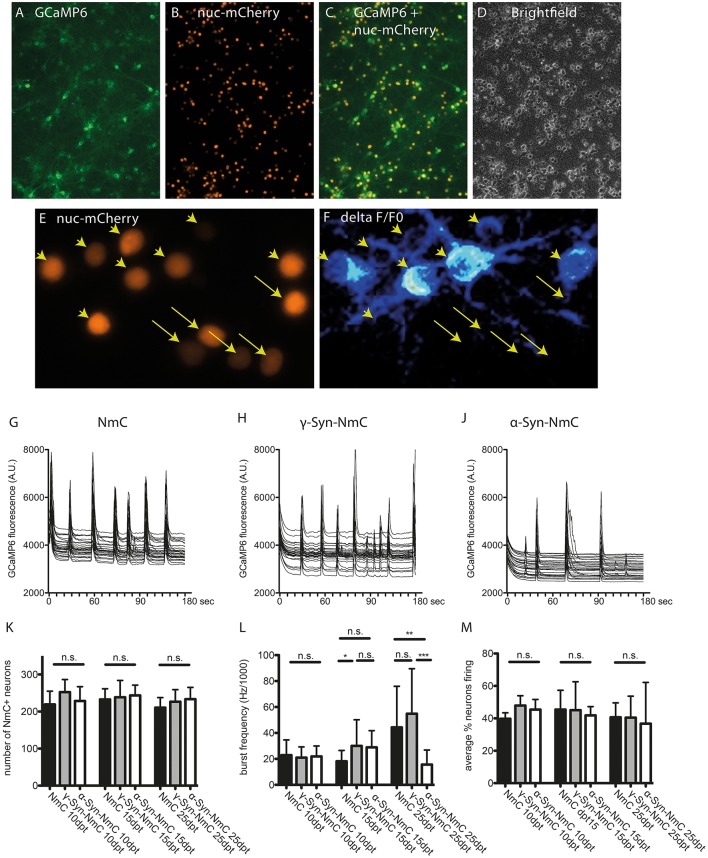
Determination of endogenous network activity of synuclein overexpressing neurons. Cultured cortical neurons were co-transduced with AAV vectors expressing the cytoplasmic Ca^2+^ sensor GCaMP6 **(A)** and nucleus-targeted mCherry (NmC) together with α-, γ-, or no synuclein **(B)**. Co-transduction efficacy was > 90% **(C,D)**. Spontaneous activity in NmC-identified neurons **(E)**, represented by Ca^2+^ transients (**F**, ratio image of ΔF/F0 of GCaMP6 fluorescence) was recorded over 3 min per visual field. Arrowheads in **(E,F)** mark neurons that contribute to network activity, while arrows mark neurons that do not contribute to network activity. Representative traces of randomly selected active neurons are shown for cells expressing only NmC **(G)**, γ-Syn + NmC **(H)** or α-Syn + NmC **(J)** at 25 days after transduction (25 dpt). Absence of any neuron loss over time that could compromise network activity is shown in (**K**, at 10, 15, and 25 dpt), the frequency of synchronized activity is shown in (**L**, at 10, 15, and 25 dpt) and the average percentage of neurons contributing to an endogenous network burst is shown in (**M**, at 10, 15, and 25 dpt). Traces show individual signals of 30 neurons each, bars show mean ± SD. Black bars, neurons expressing only NmC; gray bars, neurons expressing γ-Syn + NmC; white bars, neurons expressing α-Syn + NmC. Statistical analysis by 1-way ANOVA and Tukey's *post hoc* test. ^***^p < 0.001; ^**^p < 0.01; ^*^p < 0.05; n.s. not significant. Statistical powers in **(L)** are 0,3 for the difference between NmC and γ-Syn-NmC at dpt 15, 0.85 for difference between NmC and α-Syn-Nmc at dpt 25 and 0.99 for difference between γ-Syn-NmC and α-Syn-NmC at dpt 25.

Endogenous, non-stimulated neuronal activity was recorded by Ca^2+^ imaging with the GCaMP6 sensor. In order to facilitate segmentation (i.e., identification of individual neurons), these experiments were conducted with AAV vectors co-expressing α-Syn or γ-Syn with nucleus-targeted mCherry (NmC), or expressing nucleus-targeted mCherry alone as control (Figures 7A–D). Calcium transients were recorded in randomly chosen visual fields comprising roughly 200–250 neurons for 3 minutes and were analyzed for the percentage of neurons that show Ca^2+^ transients (i.e., are electrically active) and for the frequency with which this activity occurs. As shown exemplary in Figures 7E,F, neurons participated in endogenous network activity independent from expression levels of nuclear mCherry, which can be regarded as a surrogate marker for synuclein expression levels, as both NmC and synucleins are expressed from the same AAV vector (Supplemental Figure 1).

The endogenous neuronal activity was recorded at 10, 15 and 25 days of α-Syn or γ-Syn overexpression, representative traces are shown in Figures 7G–J for neurons expressing only NmC (G), γ-Syn + NmC (H), or α-Syn + NmC (J), as recorded at 25 dpt. At all time points we found identical neuron numbers for all groups, due to the anti-apoptotic activity of Bcl-Xl (Figure 7K). The frequency of endogenous, non-stimulated bursts increased over time, from about 7 bursts/min at 10 dpt up to 17 bursts/min at 25 dpt.

This low-frequency synchronized electrical activity is well within the range of activity determined by resting state functional MRI (rs-fMRI) for endogenous cortical network activity (Raichle, 2015), and reminiscent of the default mode network (DMN) activity. DMN is one of the major low frequency “baseline activities” of the brain, independent from external input and severed by a variety of psychiatric and neurodegenerative disorders including PD (Mohan et al., 2016).

At all times γ-Syn overexpressing neurons showed very similar activity as control cells expressing only NmC, with occasional increased frequency, which, however, was not significantly different from controls (Figure 7L). In contrast, α-Syn overexpressing neurons demonstrated a clearly reduced frequency of synchronized bursts in aged cultures, i.e., at 25 dpt (Figure 7L). Importantly, the percentage of neurons that participate in synchronized bursts was identical in all conditions (Figure 7M). Further analysis of network function showed that within the population of neurons analyzed in each area only one network is active (not shown).

These results suggest that independent from its mitochondria-related neurotoxicity, α-Syn can cause neuronal dysfunction on a systemic level.

### Summary of results

In conclusion, our results show that α-Syn overexpression in primary neurons does not induce disturbances in Ca^2+^ homeostasis, ATP production, essential electrophysiological parameters or stress kinase signaling. α-Syn overexpression causes mitochondrial deformation, increased mitochondrial thiol oxidation and apoptosis downstream of mitochondrial membrane perforation. Excitability of α-Syn overexpressing neurons is restrained, and in aged neuron cultures that were manipulated to allow for long-term survival of α-Syn overexpression, endogenous network activity is substantially diminished.

## Discussion

While compelling evidence suggests a direct contribution of α-Syn to initiation and/or progression of Parkinson's disease, the mode of neurotoxicity that this protein initiates and/or executes is still incompletely understood. Much has been learned from studies conducted in solution and cell free systems about aggregation, membrane interaction and pore formation of synucleins, and many facets of synuclein toxicity have been addressed in cellular systems. However, the vast majority of such studies were performed in conventional cell lines or immortalized brain cells, which are only neuron-like cells. Thus, it is still necessary to comprehensively verify or disprove pathophysiological mechanisms which have been proposed for α-Syn within primary mammalian neurons. It is even more important to elucidate the relative contribution of different pathophysiological mechanisms within the context of their potential interactions.

Taken together, our results suggest a surprisingly mono-causal mode of neurotoxicity exerted by α-Syn: we found mitochondrial swelling as the earliest event (Taschenberger et al., 2013), an increased level of thiol oxidation within mitochondria, an activation of caspase activity downstream of MOMP and robust neuroprotection by anti-apoptotic Bcl-Xl. In contrast, we did not detect impairments in handling of essential ions in cytoplasm or mitochondria, no changes in cell membrane or mitochondrial membrane potential, no impairments in ATP production by oxidative phosphorylation despite considerable mitochondrial deformation, no induction of canonical stress kinase activity or down-regulation of survival signals and no neuroprotection by neuroptrophic factors, calpain inhibition or enhancement of lysosomal protease activity. These findings suggest that α-Syn primarily interacts with mitochondrial components in a way that promotes escape of electrons from the respiratory chain, but does not impair ATP synthesis or ion homeostasis directly, suggesting that the mitochondrial permeability transition pore (mPTP) is not constitutively opened. Mitochondrial swelling may be caused by osmotic fluxes due to transient openings of mPTP, or due to interactions of α-Syn directly with the OMM. Permeabilization of OMM then releases pro-apoptotic factors like cytochrome C into cytoplasm, followed by canonical downstream-of-mitochondria apoptosis, although with a few uncommon features like Bad phosphorylation or release of Erk1/2 from larger signaling complexes. Importantly, independent of these directly neurotoxic mechanisms, α-Syn impairs both, neuronal excitability by exogenous stimuli and endogenous network activity, suggesting that it might cause neuronal dysfunction even without overt degeneration and loss of neurons.

### α-Syn mediated thiol oxidation

Cytoplasmic and mitochondrial redox states after α-Syn or γ-Syn overexpression were determined without any external stimulation, thus representing true steady state conditions solely influenced by the synucleins. This was achieved with the ro1GFP fluorophore, a ratiometric, non-ph dependent sensor for the thiol/disulfite status of a respective cellular compartment, as reflected by the 2GSH/GSSG redox couple or by protein-thiol proups (Dooley et al., 2004). Due to the high pKa of ro1GFP's thiol groups these are protonated at neutral pH and therefore not oxidized directly by ROS (Pouvreau, 2014). Thus, this sensor does not report the actual levels of O^−^ or H_2_O_2_, but rather the physiological consequences that result from increased ROS levels, namely the general oxidation state of thiol groups. Furthermore, the sensor reacts relatively slow, making it excellently suited to report on subcellular equilibrium conditions rather than on short-term ROS fluctuations.

The mitochondrial redox potential as reported by the sensor was about−360 to−320 mV for α-Syn and γ-Syn overexpressing neurons at dpt6, suggesting quite reducing conditions by a high NADPH/NAD+ ratio (Hanson et al., 2004). As such, mitochondria should be able to defend themselves against thiol oxidations in mitochondrial matrix, but evidently this does not prevent α-Syn – induced neurotoxicity.

The substantially increased oxidation status detected after long-term α-Syn overexpression, suggest that ROS might have caused significant pathophysiology at this time. Short-term and moderate elevations of ROS level serve important signaling functions in cells, by reversibly altering oxidation states of protein thiol groups that act as “redox switches,” similarly to protein phosphorylation (Antunes and Brito, 2017). However, a longer-term steady state of oxidizing conditions is likely to cause toxic effects by rendering proteins non-functional or damaging membrane integrity, especially the mitochondria-specific cardiolipin moiety (Kagan et al., 2005). Importantly, a decrease in mitochondrial GSH due to increased ROS production results in increased release of cytochrome C from its cardiolipin anchors within inner mitochondrial membrane (IMM) (Yin et al., 2012). Release from cardiolipin is a direct prerequisite for the ability of cytochrome C to be released into cytoplasm through a perforated OMM (Ott et al., 2002), where it eventually induces caspase activity. The relative extent of OMM perforation caused directly by α-Syn and/or oxidation of lipid components is still unclear, but prevention of α-Syn -induced cell death by Bcl-Xl clearly shows the importance of MOMP for neurodegeneration of the primary neurons (see below). This mechanism of neurodegeneration induced by α-Syn is in line with our results that mitochondrial OXPHOS seems not to be impaired by α-Syn overexpression, not even by the resulting morphological deformation, and that mitochondrial membrane potential is also unaffected at times when we measure increased oxidative states within mitochondria. Thus, for α-Syn, our data do not support the classical modus operandi for induction of neurodegeneration, initially caused by Ca^2+^ overload of mitochondria, followed by mPTP opening, mitochondrial swelling and a consecutive overproduction of ROS. Rather, a combination of ROS-induced thiol-oxidation/GSH depletion and OMM perforation appears to cause slow and low-level release of pro-apoptotic factors from inter-membrane space, while leaving intact the mitochondrial matrix compartment and the IMM-located respiratory chain.

### α-Syn mediated neurodegeneration

Permeabilization of the OMM (often referred to as MOMP, mitochondrial outer membrane permeabilization) is the step of no return for any cell, inevitably causing cellular destruction. OMM is constitutively permeable for small molecules like ATP through the VDAC channel. However, the formation of larger pores within OMM, either by pro-apoptotic proteins like Bak and Bax, by synucleins themself, or by physical processes like lipid oxidation or enhanced membrane tension after mitochondrial swelling, allow release of factors normally sequestered in the inter-mitochondrial membrane space (IMS), like cytochrome C, into cytoplasm. Here they activate caspases, the canonical cell death executioners. The anti-apoptotic Bcl family members Bcl-2, Mcl-1 and Bcl-Xl are the major watchdogs of OMM integrity, either by sequestering pro-apoptotic factors away from OMM or by preventing their ability to form pore structures (Zheng et al., 2016).

Our finding that Bcl-Xl can almost completely protect neurons from α-Syn-induced degeneration is a further support for the hypothesis, that the oxidized milieu within mitochondria caused by α-Syn overexpression initiates cell death in absence of concomitant events like breakdown of energy production or inability to handle Ca^2+^ homeostasis, i.e., without affecting the mitochondrial matrix or rendering the respiratory chain non-functional. Bcl-Xl almost completely prevented activation of caspase-3, breakdown of Erk1/2 containing signaling complexes and loss of neurons. Thus, it is evident that MOMP is of utmost importance for α-Syn-induced neurodegeneration. A direct interaction of Bcl-Xl with α-Syn seems unlikely, given the very low k_d_ for binding of Bcl-Xl to α-Syn of 2 mM in solution (as compared to a k_d_ < 10 nM for the interaction of pro- and anti-apoptotic Bcl proteins Zheng et al., 2016). Thus, Bcl-Xl does not seem to act by sequestering α-Syn away from mitochondria. This view is further supported by the facts that Bcl-Xl did not prevent enhanced mitochondrial ROS formation nor mitochondrial spherical morphology caused by α-Syn, suggesting that its major protective functionality in this paradigm is indeed prevention of MOMP. However, the relative contribution of various putative causes of MOMP, i.e., direct pore formation by synuclein itself and/or by pro-apototic factors like Bax and Bak, or physical impacts like oxidation of membrane components or enhanced membrane tension, remain to be elucidated in forthcoming studies. The same holds true for the potential impact of alpha-synuclein on the mitochondrial fusion-fission machinery.

### α-Syn does not induce disturbances in calcium handling, ATP production or stress kinase signaling

The ability of α-Syn to interact with biological membranes, and to form ion-conductive pore-like structures in artificial membranes, is well-established (Lashuel et al., 2002; Kagan, 2012). In relation to formation of ion-conductive pores it is consensus in the field, that α-Syn causes disturbances in Ca^2+^ handling that eventually lead to cell death. For intracellular α-Syn this has been shown in SY-SH5Y cells (Hettiarachchi et al., 2009), and for extracellular α-Syn applied as especially prepared pre-formed fibrils even for neurons (Pacheco et al., 2015; Angelova et al., 2016). In contrast, our present data suggest that primary neurons, which express α-Syn at levels that eventually cause neurodegeneration, are able to maintain Ca^2+^ homeostasis over both, cytoplasma membrane and mitochondrial membrane. We detected no differences in baseline Ca^2+^ levels, peak Ca^2+^ influx, and, most importantly, decay kinetics from cytoplasm or mitochondria. Thus, it seems unlikely that in these neurons any deregulation of free Ca^2+^ homeostasis takes place, which is further strengthened by intact ATP production (confirming earlier studies exploiting respiration measurements Taschenberger et al., 2013), intact mitochondrial membrane potential and unaffected basic electrophysiological parameters. Our data thus suggest that α-Syn affects Ca^2+^ homeostasis differentially in neuron-like cells vs. genuine neurons, and that intracellular α-Syn may induce different mechanisms than extracellular α-Syn that is added in form of pre-formed oligomers or fibrils. In this context it should be noted that in neuron cultures as described in this study a considerable amount of intracellularly expressed α-Syn is secreted to the cell culture supernatant (Taschenberger et al., 2013) (in principle comparable to α-Syn secretion into CSF in the brain), and still we found no indications of disturbed ion homeostasis as could be expected in case of significant breakdown of ion gradients through ion-conductive pores.

While our results show that neurons overexpressing α-Syn for prolonged periods do not suffer from disturbed Ca^2+^ homeostasis during this time, they do not preclude a contribution of Ca^2+^ to the final execution of neuronal death. Ca^2+^ imaging shows bright and saturated fluorescence of RCaMP or GCaMP sensors immediately before the cells are dying (not shown). Such signals, however, appear to be consequences of acute cellular destruction, rather than causes of induction of neurodegeneration, emphasizing the need to monitor long-lived cells for elucidation of α-Syn—mediated neurotoxicity.

The notion that α-Syn can have different effects in neurons vs. cell lines is further supported by our finding that in neurons α-Syn does not induce canonical stress kinase signaling. These results are in pronounced contrast to findings in e.g. N2A cells, where levels of phospho-Erk, phospho-p38 and phospho-JNK MAPKs were dramatically declined though α-Syn overexpression (Iwata et al., 2001), or to SH-SY5Y cells, were Erk1/2 signaling has been claimed to be an important mediator of α-Syn toxicity (Yshii et al., 2015).

### α-Syn impacts on neuronal activity

While the mode of α-Syn induced neuron death in our cellular model could be reasonably explained, α-Syn's impact on another essential neuronal functionality, namely electrical activity, is mechanistically less well characterized. α-Syn causes diminished neuronal excitability through external stimuli (i.e., field stimulation or K^+^-mediated depolarization) and a decrease in endogenous non-stimulated activity, in the absence of detectable distortion of basic electrophysiological parameters. We cannot yet describe a molecular correlate of such diminished electrical activity. None the less, our findings deliver a potential explanation for extra-nigral functional deficits in patients with synucleinopathies even in absence of overt neuron loss, due to an impact of α-Syn on basal neuronal activity.

The importance of such endogenous, non-stimulated neuronal activity can not be overestimated: most neuronal activity in the brain is due to intrinsic, external trigger-independent networks, not only in humans but also in laboratory animals (Gozzi and Schwarz, 2016; Havlik, 2017). Only about 5 % of brain energy consumption appear to be due to evoked activity associated with alterations in cerebral blood flow (Raichle, 2015). The large cortical default mode network (DMN) becomes severely impaired in its activity by several psychiatric and neurodegenerative diseases like schizophrenia, depression, Alzheimer's and Parkinson's disease (Tessitore et al., 2012; Mohan et al., 2016; Hunt et al., 2017), underscoring the importance of “baseline activity” for proper brain function.

It may be debatable if cultured neurons in a dish can be regarded as appropriate building blocks of the DMN or similar large-area networks. None the less, we consider the impact of α-Syn on synchronized activity of these neurons in their fully matured state (that might be even considered as “aged,” inasmuch this is possible for cultured neurons) to be a valuable surrogate marker for corresponding influences *in vivo*, where decades of aberrant synuclein levels, even if much lower than in our overexpressing neurons, eventually may negatively influence network activities. In the context of PD, the finding that α-Syn reduces endogenous network activity correlates well to recent results obtained in very old α-Syn overexpressing transgenic mice: in these animals it was shown by single cell electrophysiology that nigral dopaminergic neurons adopt a reduced firing rate after long-term α-Syn overexpression (Janezic et al., 2013). Thus, our cortical neuron cell culture model recapitulates features of imminent importance found in dopaminergic neurons *in vivo*, but also demonstrates that such potentially detrimental influences of α-Syn are not limited to the dopaminergic neurotransmitter phenotype. Furthermore, low-frequency endogenous activity is considered to be one of the major criteria that render neurons vulnerable to neurodegeneration in PD (Surmeier et al., 2017).

## Conclusion

Taken together, our results demonstrate that several pathophysiological mechanisms, which have been proposed to be induced by α-Syn, seem to be irrelevant in primary neurons, such as disturbances in Ca^2+^ homeostasis, impaired ATP production or stress kinase signaling. Neurotoxicity of α-Syn clearly converges on mitochondria by inducing structural deformation, increased thiol oxidation within mitochondria, and apoptosis downstream of MOMP. It is tempting to speculate that α-Syn causes a vicious cycle located between inner and outer mitochondrial membrane, in that α-Syn—induced ROS production causes release of cytochrome C from inner membrane, which in turn induces increased escape of electrons from the respiratory chain, thereby further increasing ROS levels, which then again cause further release of cytochrome C and eventually release through the outer membrane into cytoplasm.

Importantly, α-Syn appears to be able to induce neuronal network dysfunction independent from induction of neuronal degeneration. The fact that such network dysfunction occurred only in old neurons, links this phenomenon to the major risk factor for synucleinopathies, namely aging. Forthcoming studies will now have to demonstrate if different neurotransmitter phenotypes or mutations of the synucleins might modulate these impacts.

## Author contributions

JT, MB, and SK: designed the project; JT, GT, KL, MS, GS, JK, FM, and SB: performed research; JT, GT, MZ, CD, MB, and SK: analyzed and interpreted the data; JT and SK: wrote the manuscript.

### Conflict of interest statement

The authors declare that the research was conducted in the absence of any commercial or financial relationships that could be construed as a potential conflict of interest.
