# Supplementary material for: Pathophysiological Consequences of Neuronal α-Synuclein Overexpression: Impacts on Ion Homeostasis, Stress Signaling, Mitochondrial Integrity, and Electrical Activity
Source: Front Mol Neurosci. 2018 Mar 7;11:49. doi: 10.3389/fnmol.2018.00049 (PMC5845890; doi:10.3389/fnmol.2018.00049)
Supplement: Supplementary file 1 [file DataSheet1.DOCX]

Toloe et al, Supplemental Material

**S.1 Vector genome layouts of recombinant AAV viral vectors**


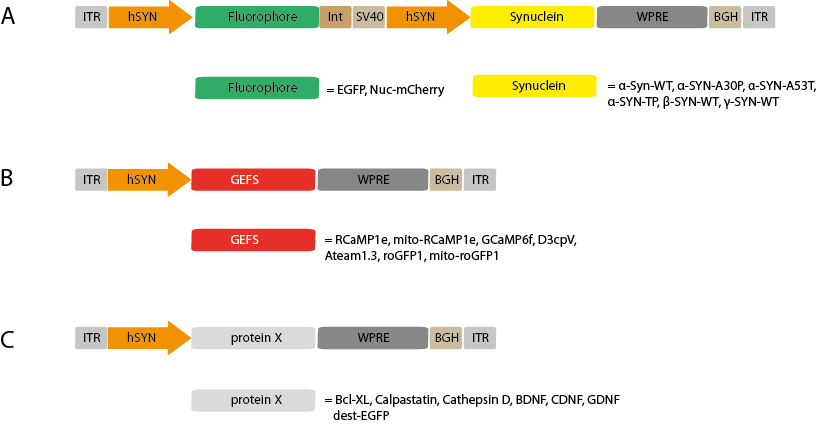


**Supplemental figure 1: AAV vector genome layouts as used in this study.**

(A) Bi-cistronic vectors for expression of various synucleins and EGFP from independent transcription units. (B) Vectors for expression of genetically encoded fluorescent sensors and (C) for proteins tested for neuroprotective potency against α-Syn neurotoxicity.

ITR=inverted terminal repeat; hSYN=neuron-specific human synapsin1 gene promoter fragment; EGFP=enhanced green fluorescent protein; Int=intron; SV40=simian virus 40 polyadenylation site; WPRE=woodchuck hepatitis virus postranscriptional regulatory element; GEFS=genetically encoded fluorescent sensors.

**S.2 Identification of protein factors counteracting α-Syn neurotoxicity**

Protein factors which could potentially inhibit different degenerative processes that might be evoked by α-Syn overexpression, were tested for their neuro-protective potency. BclXl (anti-apototic), calpastatin (inhibitor of Ca^2+^ induced calpain activity), cathepsin D (lysosomal protease postulated to degradate α-Syn) and neurotrophic factors BDNF, CDNF, GDNF (inducers of a variety of neuroprotective pathways) were thus co-expressed with aSyn and neuron numbers were quantified at indicated time points. Counting of surviving neurons was facilitated by expression of EGFP from an independent transcription unit in the AAV-synuclein vectors.


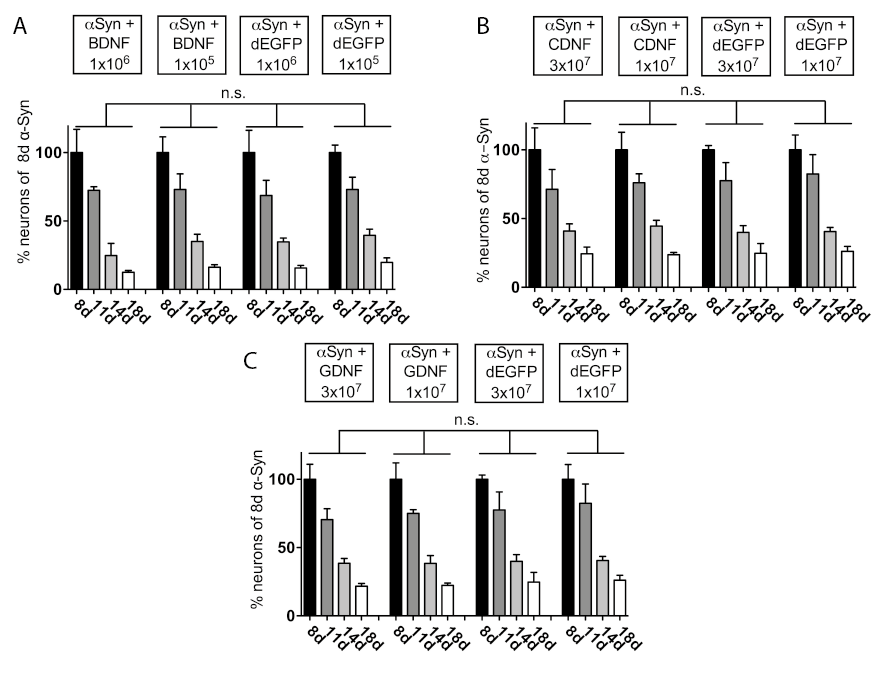


**Supplemental figure 2: Neurotrophic factors BDNF, CDNF and GDNF do not protect against α-Syn induced neurodegeneration**

Neurons were transduced with AAV-α-Syn(WT) at a titre of 1x10e8 tu/250.000 cells (MOI=400) and with

(A) AAV-BDNF at a titre of 1x10e6 tu (MOI=4) or 1x10e5 tu (MOI=0.4); higher titres of AAV-BDNF proved to be neurotoxic;

(B) AAV-CDNF at a titre of 3x10e7 tu (MOI=120) or 1x10e7 tu (MOI=40);

(C) AAV-GDNF at a titre of 3x10e7 tu (MOI=120) or 1x10e7 tu (MOI=40);

Surviving neurons were counted at days 8, 11, 14 and 18 after transduction. N.s. = not significant in 1-way Anova with Tukey´s post hoc test. N = 9 independent transductions per condition. Statistical power for not missing any significant differences at p < 0.05 is > 0.9 for all groups. Vectors expressing BDNF and GDNF have been functionally verified for neuroprotective effect in animal models recently ^1^, ^2^.


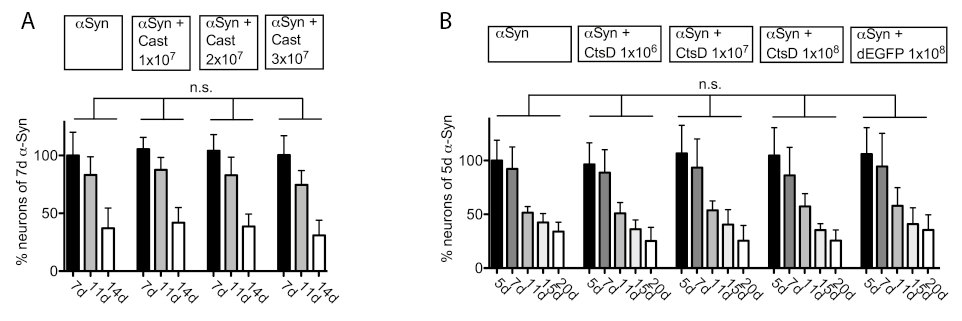


**Supplemental figure 3: Calpastatin and CathepsinD do not protect against α-Syn-induced neurodegeneration**

(A) Calpastatin does not protect from α-Syn-induced neurodegeneration. Neurons were transduced with AAV-α-Syn(WT) at a titre of 1x10e8 tu/250.000 cells (MOI=400) and with AAV-Calpastatin at a titre of 1x10e7 tu (MOI=40), 2x10e7 tu (MOI=80) or 3x10e7 tu (MOI=120). Surviving neurons were counted at days 7, 11, and 14 after transduction.

(B) Cathepsin D does not protect from α-Syn-induced neurodegeneration. Neurons were transduced with AAV-α-Syn(WT) at a titre of 1x10e8 tu/250.000 cells (MOI=400) and with AAV-Cathepsin D at a titre of 1x10e6 tu (MOI=44), 1x10e7 tu (MOI=40) or 1x10e8 tu (MOI=400) or with a control virus at 1x10e8 tu (MOI=400). Surviving neurons were counted at days 5, 7, 11, 15 and 20 after transduction.

Differences in total vector titre between groups were adjusted with a control vector expressing a destabilized EGFP without detectable fluorescence. N.s. = not significant in 1-way Anova with Tukey´s post hoc test. N = 5 – 7 independent transductions per condition. Statistical power for not missing any significant differences at p < 0.05 is > 0.9 for all groups. Vectors expressing Calpastatin and Cathepsin D have been functionally verified for neuroprotective effects in animal models recently ^3, 4^.


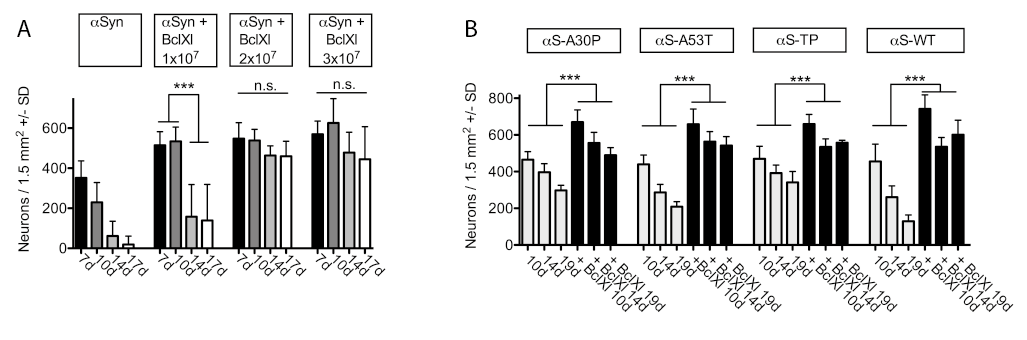


**Supplemental figure 4: Bcl-X- protects neurons from neurodegeneration induced by WT and mutant α-Syn.**

(A) Dose-dependent protection by BclXl. Neurons were transduced with AAV-α-Syn(WT) at a titre of 1x10e8 tu/250.000 cells (MOI=400) and with AAV-BclXl at a titre of 1x10e7 tu (MOI=40), 2x10e7 tu (MOI=80) or 3x10e7 tu (MOI=120). Surviving neurons were counted at days 7, 10, 14 and 17 after transduction.

B) BclXl protects from wild-type (WT) and mutant synucleins. Neurons were transduced with AAV-α-Syn(A30P), AAV-α-Syn(A53T), AAV-α-Syn(TP; “triple proline”) or AAV-αSyn(WT) at a titre of 1x10e8 tu/250.000 cells (MOI=400) and with AAV-BclXl at a titre of 2x10e7 tu (MOI=40). Surviving neurons were counted at days 10, 14 and 19 after transduction.

Differences in total vector titre between groups were adjusted with a control vector expressing a destabilized EGFP without detectable fluorescence. *** = p<0.001; n.s. = not significant in 1-way Anova with Tukey´s post hoc test. N = 4 – 7 independent transductions per condition. Statistical power > 0.95 for significant differences between groups.

**S.3 NMR-based identification of α-Syn - Bcl-XL interaction**


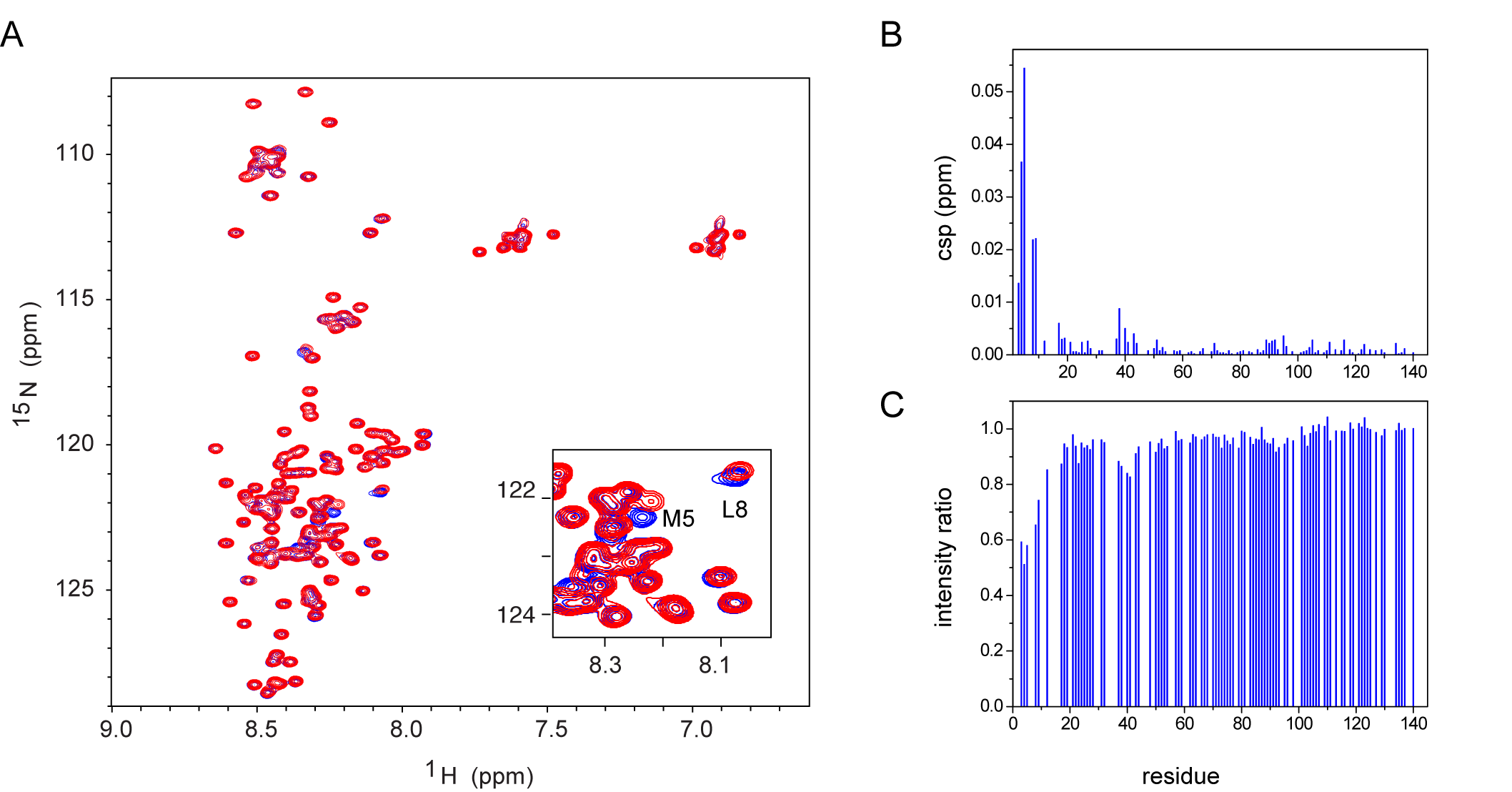


**Supplemental figure 5: Bcl-XL binds to disordered α-Syn. A.** Two-dimensional ^1^H-^15^N HSQC spectra of ^15^N-labelled alpha-synuclein in the absence (blue) and presence (red) of unlabeled Bcl-XL at 1:21 molar ratio. **B, C.** Plots of chemical shift (B) and signal intensity (C) changes from spectra in A as a function of alpha-synuclein residue number. Residues affected by signal overlap were excluded from the analysis.


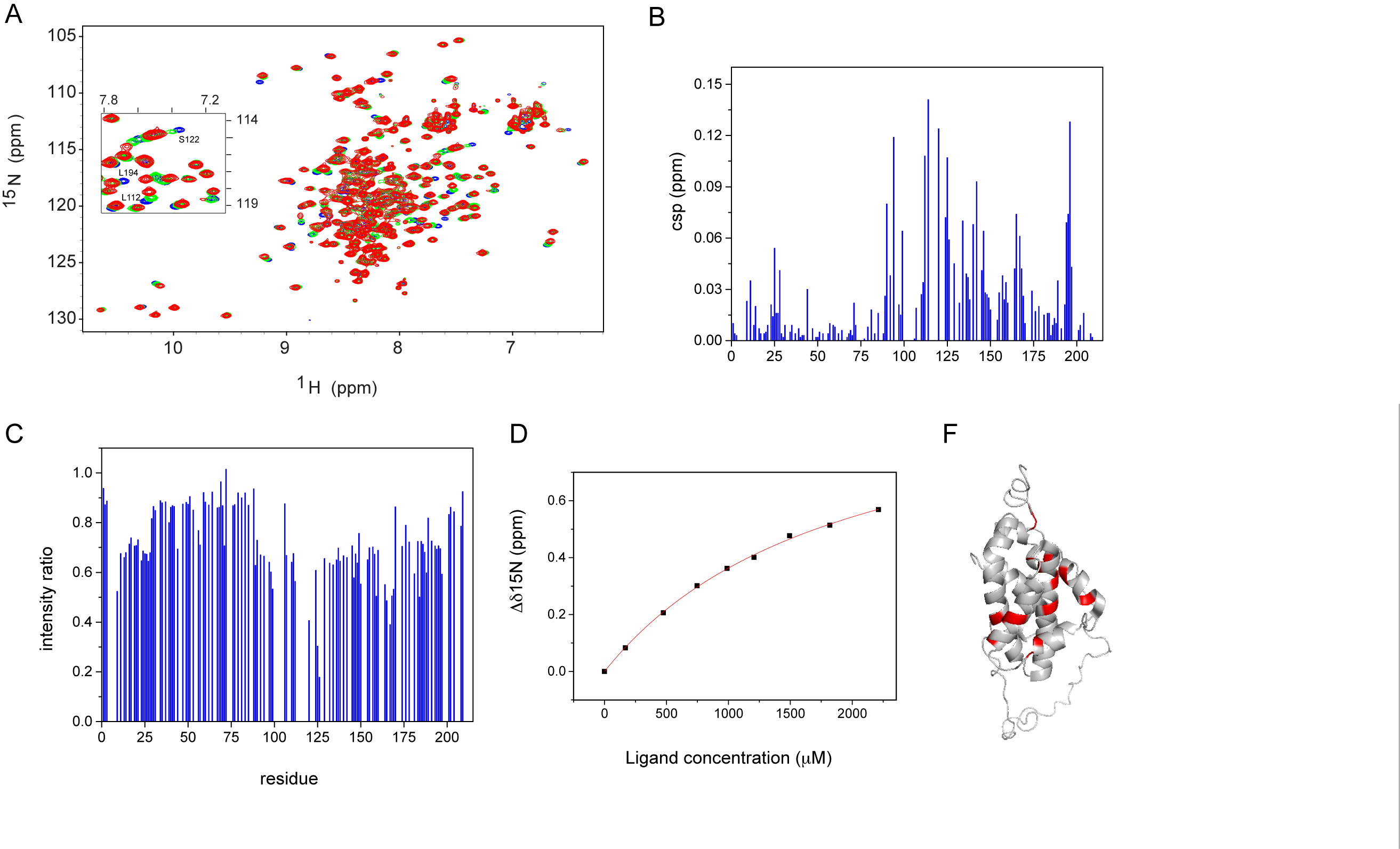


**Supplemental figure 6: Identification of the α-Syn binding site on BCl-XL.** **A.** ^1^H-^15^N HSQC spectra of ^15^N-labelled Bcl-XL in the absence (blue) and presence of unlabeled alpha-synuclein peptide 1-26 at a 1:20 molar ratio (green) or unlabeled N-terminal acetylated alpha-synuclein peptide 1-26 at a 1:20 molar ratio (red). **B, C.** Plot of chemical shift (B) and signal intensity (C) changes from ^1^H-^15^N HSQC of ^15^N-Bcl-XL in the presence of N-terminal acetylated alpha-synuclein peptide 1-26 at 1:20 molar ratio, with respect to free ^15^N-Bcl-XL. Residues affected by signal overlap were excluded from the analysis. **D.** Single-site binding model fitting of data for one of the affected residue, G94, upon titration of ^15^N-labeled Bcl-XL with N-terminal acetylated alpha-synuclein peptide 1-26. **F.** Residues showing chemical shift changes larger than 0.06 ppm were mapped in red onto the 3D structure of Bcl-XL ^5^ The gray background includes residues with shift changes smaller than 0.06 ppm and residues that could not be analyzed because of signal overlap.

**S.4 Impacts of synucleins on host cell proteins**


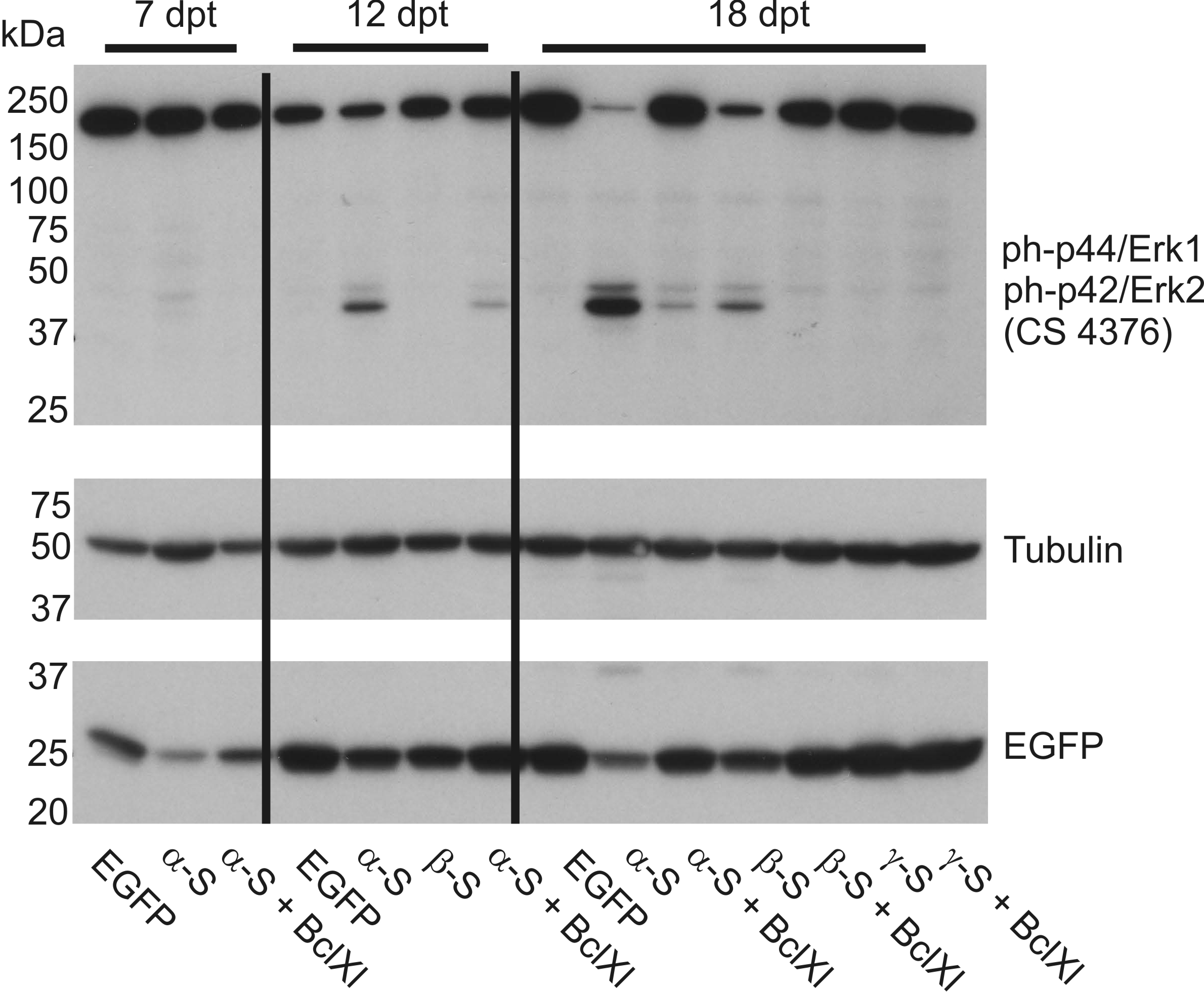


**Supplemental figure 7: Time course of α-Syn mediated liberation of phospho-Erk1/2 from a high molecular weight signalling complex**

Thr202 phosphorylated Erk1 / Thr187 phosphorylated Erk2 was detected in cell lysates obtained at 7, 12 and 18 days after transduction with AAVs expressing EGFP, α-, β-, or γ-Syn + EGFP, or α-Syn + EGFP + BclXl. Detection of tubulin and EGFP served as loading standards.


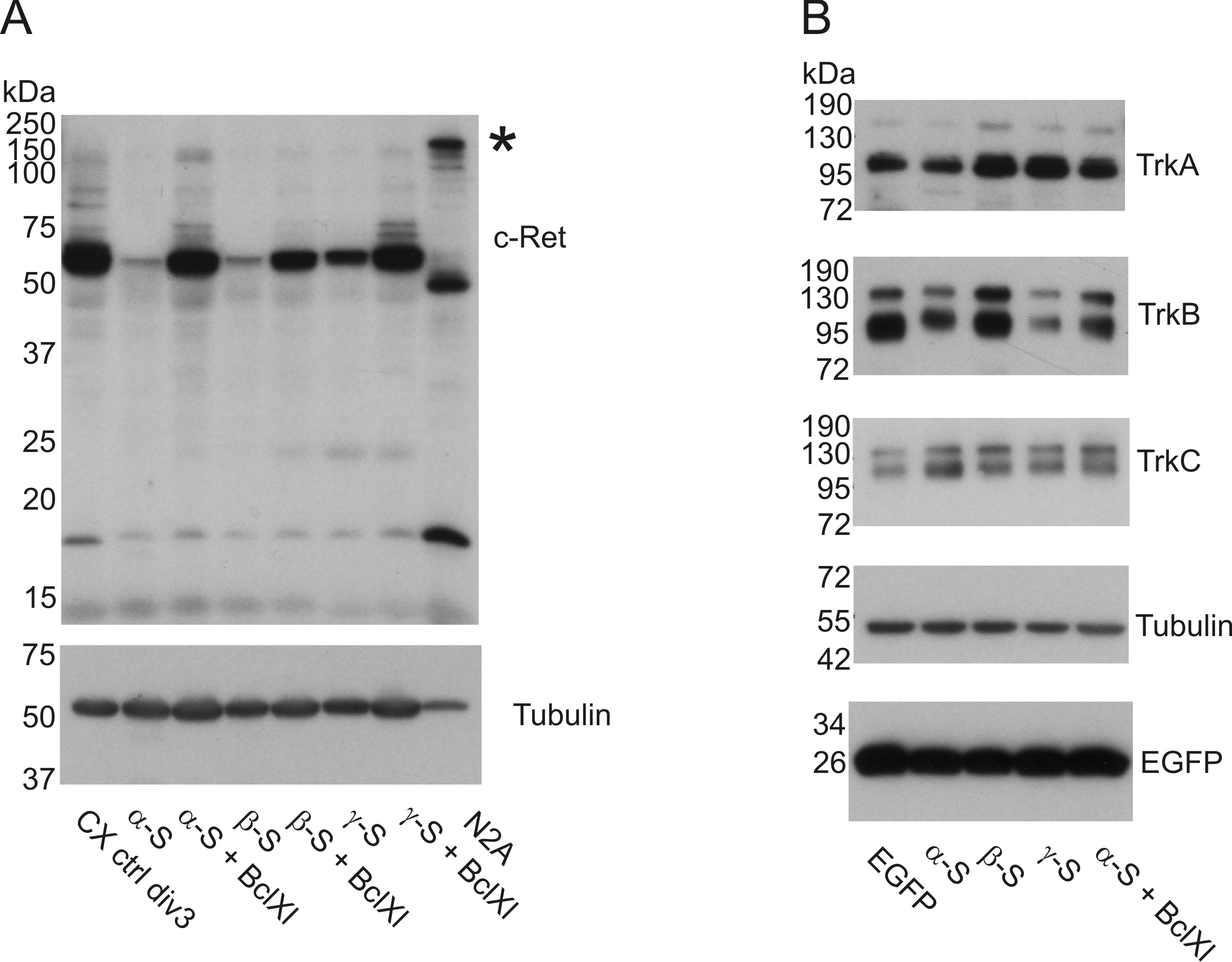


**Supplemental figure 8: α-Syn mediated downregulation of truncated Ret-receptor but not of neurotrophin receptors**

Primary cortical neurons in culture express only low amounts of full length Ret, as compared to N2A cell used as controls (A, marked by the *). The major Ret species detectable is of molecular eight 60 kDa, both in control neurons lysed at 3 days after seeding and in matured neurons at 12 days after transduction with respective synuclein expressing vectors (A). In contrast to evident downregulation of Ret through α-Syn and β-Syn (but not γ-Syn), expression levels of the neurotrophin receptors TrkA, TrkB and TrkC were unaffected by α-Syn.

**S.5 Supplemental references**

1. Ziemlinska E, Kügler S, Schachner M, Wewior I, Czarkowska-Bauch J, Skup M. Overexpression of BDNF increases excitability of the lumbar spinal network and leads to robust early locomotor recovery in completely spinalized rats. PLoS One. 2014;9:e88833

2. Shevtsova Z, Malik I, Garrido M, Schöll U, Bähr M, Kügler S. Potentiation of in vivo neuroprotection by BclX(L) and GDNF co-expression depends on post-lesion time in deafferentiated CNS neurons. Gene Ther. 2006;13:1569-1578

3. Simoes AT, Goncalves N, Koeppen A, Deglon N, Kügler S, Duarte CB, Pereira de Almeida L. Calpastatin-mediated inhibition of calpains in the mouse brain prevents mutant ataxin 3 proteolysis, nuclear localization and aggregation, relieving Machado-Joseph disease. Brain. 2012;135:2428-2439

4. Shevtsova Z, Garrido M, Weishaupt J, Saftig P, Bähr M, Lühder F, Kügler S. CNS-expressed cathepsin D prevents lymphopenia in a murine model of congenital neuronal ceroid lipofuscinosis. Am J Pathol. 2010;177:271-279

5. Follis AV, Llambi F, Ou L, Baran K, Green DR, Kriwacki RW. The DNA-binding domain mediates both nuclear and cytosolic functions of p53. Nat Struct Mol Biol. 2014;21:535-543
